# High coastal eddy activity around Antarctica revealed by SWOT

**DOI:** 10.1093/nsr/nwag181

**Published:** 2026-03-24

**Authors:** Xianxian Han, Qiang Wang, Andrew L Stewart, Zhaomin Wang, Qinghua Yang, Qinbiao Ni, Chengyan Liu, Dake Chen

**Affiliations:** School of Marine Sciences, Sun Yat-sen University, and Southern Marine Science and Engineering Guangdong Laboratory (Zhuhai), Zhuhai 519000, China; Alfred Wegener Institute, Helmholtz Centre for Polar and Marine Research (AWI), Bremerhaven 27570, Germany; Alfred Wegener Institute, Helmholtz Centre for Polar and Marine Research (AWI), Bremerhaven 27570, Germany; Department of Atmospheric and Oceanic Sciences, University of California, Los Angeles, CA 90095, USA; School of Marine Sciences, Sun Yat-sen University, and Southern Marine Science and Engineering Guangdong Laboratory (Zhuhai), Zhuhai 519000, China; School of Atmospheric Sciences, Sun Yat-sen University, Zhuhai 519000, China; School of Marine Sciences, Sun Yat-sen University, and Southern Marine Science and Engineering Guangdong Laboratory (Zhuhai), Zhuhai 519000, China; School of Marine Sciences, Sun Yat-sen University, and Southern Marine Science and Engineering Guangdong Laboratory (Zhuhai), Zhuhai 519000, China; School of Marine Sciences, Sun Yat-sen University, and Southern Marine Science and Engineering Guangdong Laboratory (Zhuhai), Zhuhai 519000, China; School of Marine Sciences, Sun Yat-sen University, and Southern Marine Science and Engineering Guangdong Laboratory (Zhuhai), Zhuhai 519000, China; State Key Laboratory of Satellite Ocean Environment Dynamics, Second Institute of Oceanography, Ministry of Natural Resources, Hangzhou 310000, China

**Keywords:** mesoscale eddies, Antarctic coastal ocean, dense overflow, ice-shelf melting

## Abstract

Antarctic marginal seas are crucial for the global climate, but direct observations, especially of mesoscale ocean eddies, remain scarce. Here, by analyzing the unprecedented high-resolution sea surface height data provided by the recently launched Surface Water and Ocean Topography (SWOT) satellite, we reveal a widespread presence of mesoscale eddies across the Antarctic continental shelf. The geographic distributions of the observed eddies, along with eddy-resolving model simulations, support the hypothesis that ice shelf basal melting and dense shelf water formation are key processes driving the prevalent eddy activity. Our findings highlight the potential of innovative satellite measurements for monitoring critical Antarctic oceanic processes, and the need to resolve the abundant Antarctic ocean eddies in climate models.

## Teaser

Using SWOT satellite, this study reveals for the first time a widespread presence of mesoscale eddies across the Antarctic continental shelf. Ice shelf basal melting and dense shelf water formation are key processes driving the prevalent eddy activity.

## INTRODUCTION

Antarctic marginal seas are characterized by a variety of dynamical processes, such as ice shelf basal melting driven by warm water intrusions [1–3], and dense shelf water (DSW) formation resulting from brine rejection during strong sea ice production in coastal polynyas [4,5]. These processes, distributed across the Antarctic marginal seas, are crucial for global overturning circulation, sea level rise, ocean stratification, and carbon sequestration [6–9]. Mesoscale eddies are critical to regional and global processes in the climate system [10,11], and may thus be anticipated to play similarly important roles in Antarctic shelf processes. However, the occurrence, characteristics and role of mesoscale eddies over the Antarctic continental shelf remain poorly understood [12,13]. This knowledge gap arises from the challenges of observing and modeling mesoscale oceanic eddies in these remote and complex regions [14,15].

Both ice shelf melting and DSW formation create cross-shelf density gradients, which may be anticipated to lead to an energetic mesoscale eddy field via hydrodynamic instabilities [16,17]. However, *in situ* observations in Antarctic marginal seas have mainly relied on ship cruises and moorings [17–23]. These observations are limited to specific sites and lack the spatial-temporal coverage necessary to fully describe the pan-Antarctic mesoscale oceanic processes [24–27]. In contrast, satellite observations can survey oceanic flows across the entire Antarctic margins, provided that those flows exhibit substantial surface signals. Satellite data have been widely used to study large-scale and mesoscale ocean circulations in the broader Southern Ocean [28–31]. However, traditional altimetry provides only along-track one-dimensional sampling, and multi-satellite combined sea surface height (SSH) data are too coarsely spaced (∼25 km) to resolve mesoscale processes in Antarctic marginal seas [30], where the Rossby deformation radius is typically *≲*5 km [32–34]. Similarly, studies using numerical simulations are limited by the small scales of Antarctic shelf mesoscale features, typically focusing on specific areas of the continental shelf [34–37]. The lack of an observational complement to such studies leaves a critical gap in our understanding of mesoscale eddies around the Antarctic continental shelf.

The recently launched Surface Water and Ocean Topography (SWOT) satellite marks a major advancement over traditional altimeters by providing a two‐dimensional SSH product, with relatively fine (2 km) grid spacing [38,39]. This resolution is comparable to the Rossby deformation radius (∼2–5 km) [33,34] in Antarctica’s marginal seas, suggesting that we can study mesoscale processes and estimate flow speed and direction through geostrophic balance. This advance in observational capability is therefore analogous to the advent of traditional altimetry for studying mesoscale eddies in the open ocean, as the resolution of traditional altimetry is comparable to the deformation radius there [32,40]. With a single SWOT swath spanning 50 km in width, this data can resolve eddies with radii ranging from ∼2 to 25 km, which cover the scale of mesoscale eddies expected in Antarctic marginal seas [33,34]. This capability offers an unprecedented opportunity to comprehensively characterize pan-Antarctic surface mesoscale eddies, providing novel insights into the dynamical processes occurring around the Antarctic coast.

In this study, using SWOT observations, we present the discovery of abundant mesoscale eddies around the Antarctic continental shelf, and describe their statistical features. We find that these eddies are prevalent near rapidly melting ice shelves and DSW formation regions. Complementary high-resolution idealized simulation experiments support our hypothesis that both ice shelf meltwater plumes and DSW formation in coastal polynyas contribute to the generation of these eddies.

## HIGH EDDY ACTIVITY OBSERVED BY SWOT

**Figure 1.**
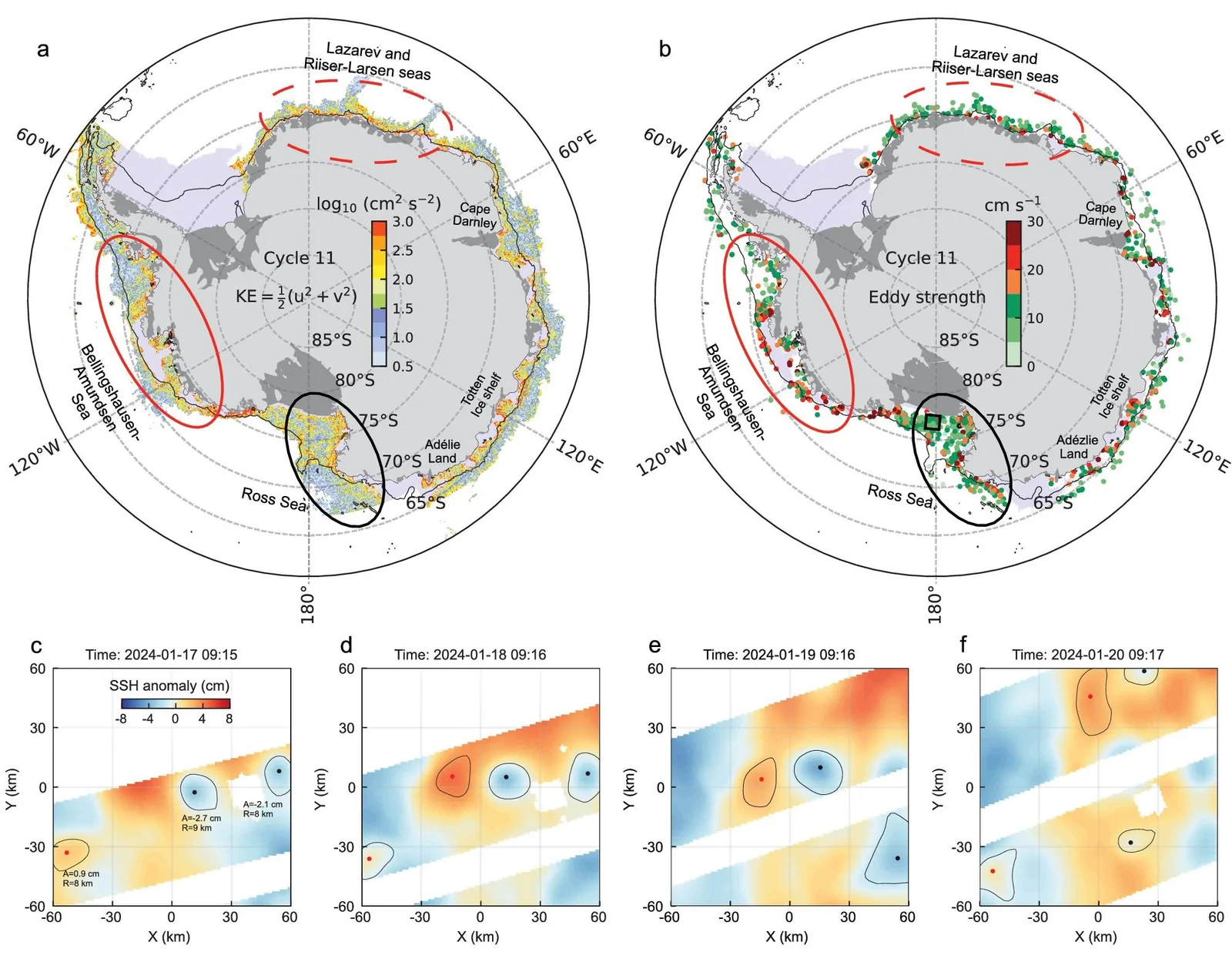
SWOT observations. (a) Snapshot of kinetic energy (KE) from cycle 11 (14 February–6 March 2024). (b) Eddies identified by closed contours of sea surface height (SSH) anomaly accumulated over the 21-day cycle, with color shading representing eddy geostrophic rotational speed (Equation 1). Only eddies with SSH anomaly amplitudes exceeding 1 cm are shown. Black and light purple shading indicate ice shelf and sea ice coverage, respectively. Areas with sea ice concentration >40% are masked (see Methods). The red and black ellipses mark regions in which ice shelf meltwater and DSW formation are expected to dominate cross-shelf density gradients, respectively. The red dashed ellipse indicates the region with strong Antarctic Slope Current. The circum-Antarctic black line indicates the 1000 m isobath. Open ocean areas are masked (see Methods). (c–f) Daily SSH anomaly evolution in the southern Ross Sea (black rectangle in panel b) from SWOT cycle 09. Black circles denote eddy boundaries, while black and red dots mark the centers of cyclonic and anticyclonic eddies, respectively. Panel c illustrates examples of eddy amplitude (A) and radius (R). The data are projected from geographic coordinates onto Cartesian distance coordinates, with the domain centered at 76.8°S, 179.8°W.

Daily eddy tracking reveals that the geostrophic length scale can indeed be as small as ∼5 km (Fig. 1 and Fig. S1), comparable to the spatial resolution of SWOT data. Given that the flows are expected to be approximately in geostrophic balance, and this enables the estimation of horizontal near-surface flow from the SSH gradient:


(1) \begin{eqnarray*}
\!\!\! u = - \frac{g}{f}\frac{{\partial \eta }}{{\partial y}},\,\, v = \frac{g}{f}\frac{{\partial \eta }}{{\partial x}},\,\, V = \ \left| {\frac{g}{f}\frac{{\Delta \eta }}{{\Delta r}}} \right|.
\end{eqnarray*}

Here, $( {u,v} )$ are the zonal and meridional geostrophic velocity, respectively, *g* is the gravitational acceleration, *f* the Coriolis parameter, *η * the absolute dynamic height, *V* the eddy strength, and $\Delta \eta $ and *Δ r* are eddy amplitude (the SSH difference between eddy center and edge) and radius (see Methods), respectively. From these estimates, we calculate the kinetic energy (KE) associated with the geostrophic velocity (Fig. 1a). Since each SWOT pass observes two 50 km-wide swaths (Fig. 1c–f), achieving full coverage of Antarctica requires multiple passes over the ∼21-day orbital cycle. Due to swath overlap during this period (see Methods and Fig. S1), only the most recent swath is shown in Fig. 1a.

**Figure 2.**
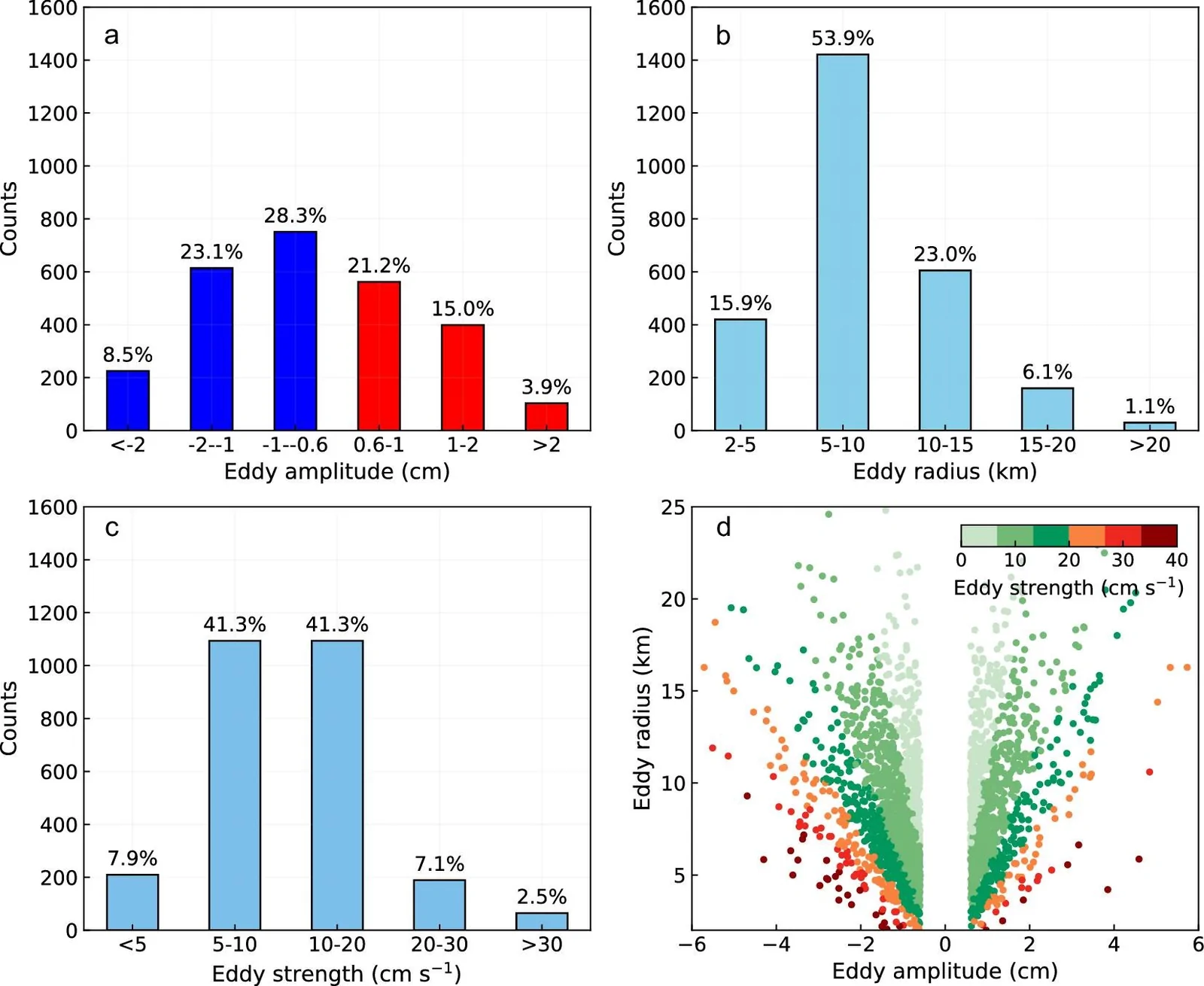
Eddy statistical characteristics around Antarctica’s coast. (a) Eddy amplitude, defined as the sea surface height difference between eddy center and edge (center minus edge), with negative values corresponding to cyclonic eddies. (b) Eddy radius. (c) Eddy strength, defined by a bulk estimate of the eddy geostrophic flow speed (Equation 1). (d) Relationship between eddy amplitude, radius, and strength. Eddies with absolute amplitudes <0.6 cm are excluded to minimize the influence of measurement uncertainty on the statistical characteristics (see Methods).

SWOT SSH data are limited by sea ice coverage [41], so we focus on cycle 11 (14 February to 6 March 2024), during which pan-Antarctic sea ice extent is near its annual minimum (other available cycles are shown in Fig. S2). Figure 1a shows that KE along the Antarctic margins is substaintially stronger than in offshore regions and exhibits pronounced spatial variability. To validate that the strong KE is associated with activity of mesoscale eddies, we further identify eddies using closed SSH anomaly contours [32,42]. Specifically, we detect eddies in each swath and aggregate them across a SWOT cycle (see Methods). The results reveal a spatial distribution of eddies (Fig. 1b) consistent with the KE spatial pattern, supporting our assertion that the KE distribution is primarily driven by mesoscale eddies. To provide a quantitative understanding of the observed eddy characteristics, we analyze the features of the detected eddies from this SWOT cycle around the Antarctic marginal seas (Fig. 2).

Our analysis reveals that the total number of cyclonic eddies (rotating clockwise, 60%) exceeds that of anticyclonic eddies (rotating counter-clockwise, 40%) (Fig. 2a), consistent with the positive skew in the vorticity probability density distribution toward cyclonic values (Fig. S3). Such a cyclonic dominance could be explained by the well-known cyclone-anticyclone asymmetry that occurs, in part, because anticyclonic eddies are unstable under strong rotation [43]. Most of the eddy amplitudes fall within 2 cm (Fig. 2a), and over half of the detected eddies have radii between 5 and 10 km (Fig. 2b). These results are consistent with the small Rossby deformation radius characteristic of the Antarctic marginal seas [33,34]. By combining eddy amplitude and radius, the estimated eddy strength is concentrated in the range of 5–20 cm/s (Fig. 2c). Figure 2d illustrates the relationship between the eddy amplitude, radius, and strength across the entire population of detections.

Although eddies are widespread around the Antarctic margins, their distribution exhibits significant spatial variability. For example, strong and concentrated eddy activity is observed in the Bellingshausen–Amundsen Sea and the Ross Sea (Fig. 1b), where the Antarctic Slope Current (ASC) is relatively weak or absent [15], allowing the dominant eddy generation mechanisms to be more clearly distinguished. The ice shelf cavities in the Bellingshausen–Amundsen Sea sector exhibit the largest area-averaged basal melting rates of all Antarctic ice shelves [1,3] (Fig. S4), potentially generating strong mesoscale eddies through outflowing meltwater plumes [44]. In the Ross Sea, the abundance of eddies aligns with the pathways of DSW export [19,45], suggesting that the latter leads to formation of eddies with detectable surface expression [36,46]. Ice shelf melting and DSW formation are also prevalent in East Antarctica and may therefore contribute to eddy activity there [1,4]. In contrast, instabilities of the ASC [15,47] may play an important role in eddy generation in regions such as the Lazarev and Riiser-Larsen Seas, where strong KE is concentrated along the 1000 m isobath, indicating an intensified ASC (Fig. 1a). The locally enhanced meltwater input from the widespread ice shelf cavities in the Lazarev and Riiser-Larsen Seas [48,49] (Fig. S4) may also contribute.

In summary, observations lead us to hypothesize that both ice shelf melting and DSW formation are key drivers of surface eddy activity along Antarctic margins. In the following sections, we test this hypothesis via targeted model simulation experiments.

## EDDIES GENERATED BY ICE SHELF MELTWATER EXPORT

To better represent the distribution and rotation of eddies, we define the vortex Rossby number (${R}_{{o}_v}$) as


(2) \begin{eqnarray*}
{R}_{{o}_v} = \frac{\xi }{f},
\end{eqnarray*}

where $\xi = \frac{{\partial v}}{{\partial x}} - \frac{{\partial u}}{{\partial y}}$ is the relative vorticity [50]. The calculated ${R}_{{o}_v}$ are similar whether using simulated surface velocity or surface geostrophic velocity derived from simulated SSH in our model (Fig. S5), with a root mean square deviation of 0.02, corresponding to 20% of the spatial standard deviation (see Methods). This supports our approach of calculating the ${R}_{{o}_v}$ from the SWOT-observed SSH via geostrophic balance.

**Figure 3.**
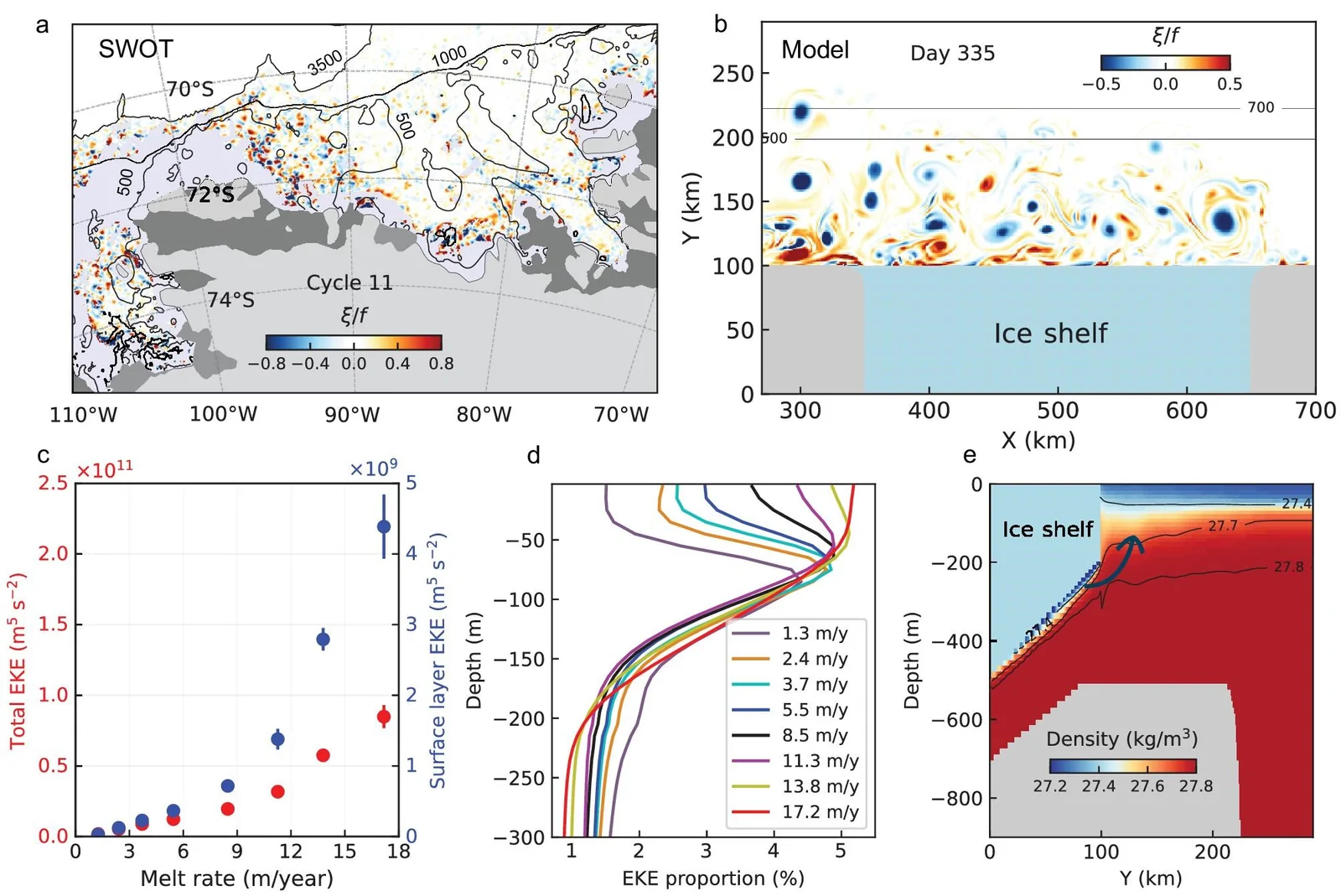
Observations and numerical experiments for eddies induced by ice shelf melting. (a) Snapshot of vortex Rossby number in the Bellingshausen–Amundsen Sea from SWOT observations (14 February–6 March 2024). Black and light purple shading indicate ice shelf and sea ice coverage, respectively. (b) Snapshot (daily mean) of surface vortex Rossby number in an experiment with an area-averaged ice shelf melt rate of 11.3 m per year. (c) Spatially integrated EKE vs. ice shelf melt rate. EKE is calculated for the entire water column (red dots) and the surface layer (defined as the upper 10 m of the water column, blue dots), excluding the ice-shelf cavity. Values are averaged over the last 180 days (Fig. S7), with the error bars representing the temporal standard deviation. (d) EKE vertical distribution for each 10-m-thick layer, normalized by total EKE in the corresponding experiment (see Methods). (e) Cross-section of zonally averaged potential density (referenced to 0 dbar), with the horizontal extent indicated in panel b.

SWOT observations in the Bellingshausen–Amundsen Sea reveal vigorous mesoscale or even submesoscale eddy activity [51] (Fig. 3a), with spatial patterns that closely follow strong lateral density gradients [52]. To elucidate whether ice shelf melting alone can lead to the generation of surface eddies in the vicinity of ice shelf cavities, we conducted idealized numerical model simulations (see Methods). These simulations use an initial vertical stratification representative of typical Antarctic coastal regions (Fig. S6), while assuming horizontally uniform density to initialize the ocean in a resting state. The ice shelf is 200 m thick at the front, gradually thickening to 500 m at the southern boundary (Fig. 3e).

The model results indicate the presence of strong surface eddies, supporting the hypothesis that meltwater plumes alone can trigger eddy generation [17,53]. To examine the influences of different melt rates, we conducted a series of sensitivity experiments, varying the ice-shelf basal drag coefficient while keeping all other aspects of the model configuration unchanged. The results show that the total eddy kinetic energy (EKE, see Methods) increases super-linearly with melt rate, with a more pronounced increase in the surface layer (Fig. 3c). The latter is further illustrated by the normalized vertical distribution of EKE (Fig. 3d), which indicates that stronger melt rates result in EKE becoming more concentrated in the surface layer, making it more easily detectable via satellite observations. This phenomenon can be interpreted as follows: under the conditions of weak melting, the meltwater plume mixes with denser ambient water before it can upwell to the surface. Consequently, the baroclinic instability-induced eddies are concentrated at the pycnocline depth (∼100 m; see Fig. 3d–e and ref. [22,54]). In contrast, stronger melting generates a larger volume of meltwater, facilitating upwelling to shallower depths and producing stronger eddies in the surface layer. These findings suggest that satellite altimetry could potentially be used to monitor ice shelf meltwater discharge indirectly, particularly given the decrease in sea ice cover and increase in ice shelf melting projected under future climate change [55].

## EDDIES GENERATED BY DSW FORMATION

In the Ross Sea, intensified sea ice formation and the associated brine rejection in polynyas lead to formation of DSW, which resides on the continental shelf and is exported offshore primarily through the Drygalski Trough (DT) and Glomar Challenger Trough (GCT) [45]. Theoretically, the density difference between DSW and its ambient waters can induce baroclinic instability, leading to the generation of mesoscale eddies [36,56,57]. However, direct observational evidence for this process at such small scales remains lacking.

**Figure 4.**
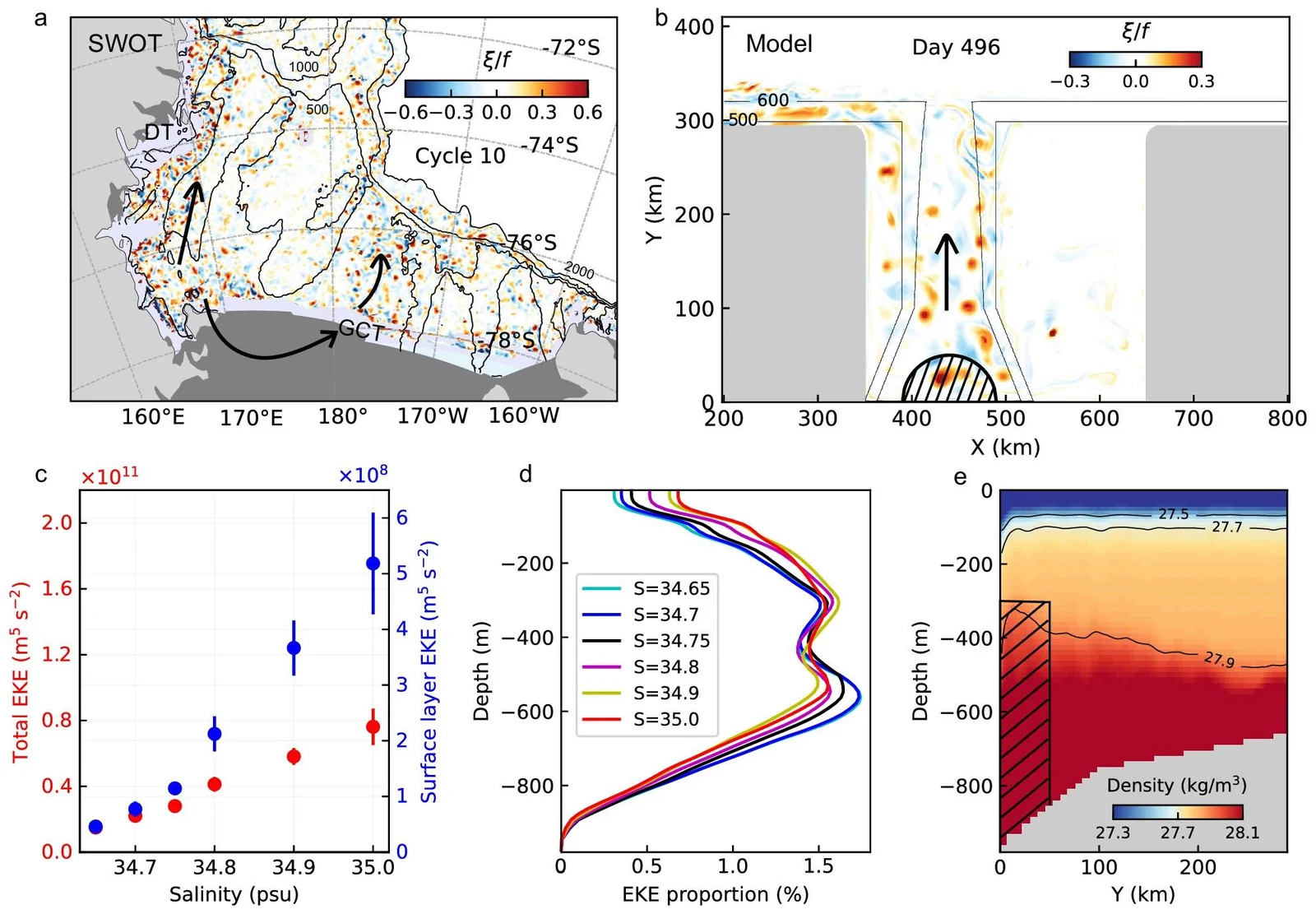
Observations and numerical experiments on eddies generated by DSW formation and export. (a) Snapshot of vortex Rossby number in the Ross Sea from SWOT observations (25 January–14 February 2024). Black arrows indicate the approximate export pathways of DSW [45,58]. DT: Drygalsky Trough; GCT: Glomar Challenger Trough. Black and light purple shading indicate ice shelf and sea ice coverage, respectively. (b) Snapshot (daily mean) of surface Rossby number in an experiment with DSW salinity of 35 psu. The region with tilted lines near the southern boundary of the trough indicates the location where dense water restoring is applied. (c) Spatially integrated EKE vs. salinity. EKE is calculated for the entire water column (red dots) and the surface layer (10 m thick, blue dots). Values are averaged over the last 200 days (Fig. S8), with the error bars representing the temporal standard deviation. (d) EKE vertical distribution for each 10-m-thick layer, normalized by total EKE in the corresponding experiment (see Methods). (e) Cross-section of zonally averaged potential density (referenced to 0 dbar), over the horizontal extent shown in panel b. Note that the spatial region analyzed in panels c–e is confined to the trough area.

The snapshot of SWOT observations shows that a high density of eddies with relatively high vortex Rossby numbers are found in both the DT and GCT (Fig. 4a), closely aligning with the pathways of DSW export [45]. This suggests that DSW formation and its export can lead to generation of mesoscale eddies, whose flows extend to the surface. To further investigate this, we conducted an idealized model experiment excluding the ice shelf but incorporating a large embayment and a trough (Fig. 4b). Similar to previous studies [46,48,59], dense water was restored at the southern boundary of the trough to mimic the formation of DSW, serving as the only driver of oceanic motions in our model. Note that we only restore DSW at depths below 300 m (Fig. 4e), so any surface eddies present can be unambiguously attributed to DSW from the ocean interior.

The model results reveal a concentration of surface eddies within the trough on the continental shelf (Fig. 4b), providing evidence that the export of DSW can indeed produce surface eddy signals. To assess the sensitivity of this result, we performed a series of experiments varying the salinity of DSW (see Methods). These sensitivity experiments reveal that both total and surface-layer EKE increase nearly linearly with increasing DSW salinity (Fig. 4c). While EKE becomes more concentrated in the surface layer for denser DSW, its maximum consistently occurs in the ocean interior and decays toward the surface (Fig. 4c and d). These results further support our hypothesis that the density gradients associated with DSW outflow drive the generation of mesoscale eddies. Although eddy strength weakens toward the surface, the presence of surface signals suggests that satellite altimetry may be used to monitor DSW production and export indirectly via the eddy field.

## SUMMARY AND DISCUSSION

The relatively small Rossby deformation radius (∼2–5 km) around the Antarctic marginal seas has heretofore hindered both observations and numerical simulations of mesoscale eddies, leaving a major gap in community understanding of Antarctic continental shelf processes [15,17]. In this study, we reveal the pan-Antarctic abundance and characteristics of eddies by employing SWOT observations. Statistics drawn from the population of observed eddies show that ∼70% of the eddies have a radius smaller than 10 km. Similar results are found in regional statistics (Fig. S9), as well as in pan-Antarctic statistics across multiple cycles (Fig. S10). Model-derived statistics are generally consistent with the observations, although eddy radii tend to be slightly larger with the idealized model configurations (Fig. S11). Strong eddy activity is found to be co-located with regions in which ice shelf meltwater is discharged (for example, the Bellingshausen–Amundsen Sea) or DSW is formed and exported (for example, the Ross Sea), suggesting that the cross-shelf density gradients resulting from these processes serve as the source of the eddy energy. This mechanism is supported by a series of idealized numerical experiments, which show that eddies become more concentrated in the surface layer as ice shelf melting and DSW density increase, implying potential applications of SWOT for monitoring changes in ice shelf melting and DSW export [60]. Ongoing sea ice decline, resulting in larger ice-free area, will facilitate satellite observations of eddies around the pan-Antarctic marginal seas, providing improved insights into their changes and impacts.

Ice shelf melting and DSW formation are widespread around Antarctica [1,4] and may therefore also play an important role in eddy activity in other regions along the Antarctic margins (Fig. 1b). For instance, strong eddies are observed near the Totten Ice Shelf (Fig. 1), where the melt rate is high (Fig. S4). Similarly, the impact of DSW on eddy activity is likely manifested in other typical DSW formation regions, such as Adélie Land and Cape Darnley (Fig. S2), where some DSW may persist into early summer [20,21]. Additionally, eddy characteristics also vary significantly across different SWOT cycles (Fig. S12), potentially reflecting eddy evolution (Fig. S1) as well as temporal variability in DSW export and ice shelf meltwater upwelling. To fully assess these variations, a more rigorous evaluation of SWOT measurement precision is needed to determine how accurately changes in the eddy field can be quantified.

It is noteworthy that ice shelf melting and DSW export may interact in specific regions, though we simulated them separately for clarity. In the Ross Sea, for example, strong eddies in the GCT (Fig. 4a) coincide with a pathway of ice shelf meltwater export in austral summer [23,58], suggesting that meltwater-induced eddies may also enhance eddy abundance there. In East Antarctica, instabilities of the ASC [47,49] may coincide with both ice shelf melting and DSW formation, jointly influencing local eddy activity. Other potential mechanisms, such as freezing and melting at the sea ice edge [30,61], were not examined in this study, yet they could play an important role during seasonal transitions. Quantitatively disentangling the relative contributions of different processes to eddy generation warrants further research, and will be essential for practically applying new-generation satellite measurements such as SWOT to monitor various ocean processes around Antarctica.

Eddies detected through closed SSH anomaly contours may contain some contamination from internal waves, which are primarily generated by tides and high-frequency wind forcing [62]. However, diurnal internal tide generation is suppressed at high Antarctic latitudes due to their location poleward of the critical latitude (∼30°) [63]. While semidiurnal tides may generate internal tides over the Antarctic continental shelf, their weak amplitudes (Fig. S13) suggest a minimal contribution. Notably, most of the Ross Sea continental shelf lies poleward of the semidiurnal critical latitude (∼74.5°), further suppressing internal tide generation. In addition, the persistence of eddy hotspots (Fig. S12), in contrast to surrounding low-activity regions, suggests little influence from wind-driven internal waves. Therefore, the observed SSH signals are likely to be primarily caused by geostrophic eddies. Nevertheless, further work is required to fully assess the dynamical composition of the observed SSH signals.

Uncertainties remain in the SWOT product due to the complex Antarctic coastal environment and potential geodetic errors [64], which may introduce eddy-like artifacts and warrant further investigation. Despite these limitations, the pronounced variations in vorticity (Fig. S14) and KE (Fig. S15) over successive SWOT cycles suggest the impact of these uncertainties remains limited. Moreover, the spatial coincidence between eddy hotspots and elevated chlorophyll-a and phytoplankton carbon concentrations (Fig. S16) provides independent evidence, and also suggests that eddies may play an important role in biogeochemical processes [16,53], with broader implications for Antarctic ecosystems. Sea ice limits SSH observations to the austral summer, constraining assessment of seasonal variability. Even during summer, it obscures some of the circum-Antarctic regions, especially in the Weddell Sea, thus limiting a comprehensive understanding of the eddy field. In addition, our calculations are based on the assumption of geostrophic balance, which may introduce bias in regions with elevated vorticity. Finally, SWOT observes only the surface expression of eddies. Although our idealized model offers some insight into their dynamics, future work should incorporate realistic high-resolution modeling and interior ocean observations to investigate their full three-dimensional structure and impacts.

## METHODS

Detailed descriptions of methods are presented in the Supplementary materials.

## Supplementary Material

nwag181_Supplemental_File

## Data Availability

The SWOT L3 Version 3.0 dataset is cited in reference [65]. Sea ice concentration data are derived from SWOT L2 dataset and are publicly available from AVISO+ at: https://tds-odatis.aviso.altimetry.fr/thredds/catalog/dataset-l2-swot-karin-lr-ssh-validated/catalog.html. The Chlorophyll-a and phytoplankton carbon data used in this study are publicly available from NASA’s Ocean Biology Distributed Active Archive Center (OB.DAAC) at: https://oceandata.sci.gsfc.nasa.gov/directdataaccess/. The MATLAB code for eddy detection, the MITgcm model configuration files, and the MATLAB code for generating input files are available at: https://zenodo.org/records/14735345.
