# Supplementary material for: High coastal eddy activity around Antarctica revealed by SWOT
Source: Natl Sci Rev. 2026 Mar 24;13(9):nwag181. doi: 10.1093/nsr/nwag181 (PMC13159728; doi:10.1093/nsr/nwag181)
Supplement: nwag181_Supplemental_File [file nwag181_supplemental_file.docx]

Supplementary Materials for

**High coastal eddy activity around Antarctica revealed by SWOT**

Xianxian Han^1,2^, Qiang Wang^2^, Andrew L. Stewart^3^, Zhaomin Wang^1^, Qinghua Yang^4,1^, Qinbiao Ni^1^, Chengyan Liu^1^, Dake Chen^1,5^*

1 School of Marine Sciences, Sun Yat-sen University, and Southern Marine Science and Engineering Guangdong Laboratory (Zhuhai), Zhuhai, 519000, China

2 Alfred Wegener Institute, Helmholtz Centre for Polar and Marine Research (AWI), Bremerhaven, 27570, Germany

3 Department of Atmospheric and Oceanic Sciences, University of California, Los Angeles, 90095, California, USA

4 School of Atmospheric Sciences, Sun Yat-sen University, Zhuhai, 519000, China

5 State Key Laboratory of Satellite Ocean Environment Dynamics, Second Institute of Oceanography, Ministry of Natural Resources, Hangzhou, 310000, China

Corresponding author: dchen@sio.org.cn

**The PDF file includes:**

Methods

References [66-79]

Figs. S1 to S16

**Methods**

**Processing of SWOT data:**

The SWOT satellite sampling provides high spatial resolution measurements over two 50 km-wide swaths, separated by a 20 km gap along the satellite's ground track, where data are collected by a conventional nadir altimeter (i.e., a downward-looking radar altimeter measuring directly beneath the satellite) [38]. The full SWOT repeat cycle for global coverage spans approximately 21 days. Because of the swath coverage, the number of repeat observations at a given location within this 21-day cycle varies with latitude, with higher repetition at high latitudes (Fig. S1A). For the snapshot of surface KE (Fig. 1A) and vortex Rossby number (Fig. 3A and 4A), we present data from the latest swath of a cycle, with earlier swaths overlaid. As a result, the data represent a true snapshot, but the observation times vary spatially. However, for statistics of mesoscale eddies, we count all the detected eddies over a 21-day cycle, during which the Antarctic continental shelf is sampled multiple times due to the swath coverage. Thus the eddy counts should not be interpreted as a total number of eddies observed at a specific time, but rather a running count over repeat sampling. A caveat of this approach is that there are spatial variations in the number of repeat samples within each cycle (Fig. S1A), which introduces a bias in favor of more poleward areas of the Antarctic continental shelf, such as the southern Ross Sea.

The data product we used is the SWOT_L3_LR_SSH_Expert, on a 2 km grid, version 3.0 [65]. Due to sea ice coverage, our analysis focuses on the austral summer from January to March 2024, corresponding to SWOT cycles 09 to 12. The SSH anomaly has undergone essential corrections, including the removal of geocentric ocean tides and phase-locked internal tides (provided in the SWOT datasets), which together account for the total tidal contribution, as well as other standard calibration procedures. Although some uncertainties remain in the tide correction, tidal signals are typically large scale with weak horizontal gradients and are therefore expected to have only a minor impact on the detection of small mesoscale eddies. Consistent with this, the tidal SSH components, subtracted from the SWOT observed absolute SSH, exhibit negligible vorticity compared to the mesoscale eddies identified in this study, indicating that the detiding process does not introduce significant errors to the vorticity calculation. In the Antarctic coastal region, errors in the mean sea surface product may affect the SSH anomaly (*64*), potentially leading to spurious domes or depressions that could be misidentified as stationary eddies. Although our results suggest that this effect is not significant, as the detected eddies and KE show clear temporal variability (Fig. S1, Fig. S14 and S15), further work is needed to assess potential contamination from these geodetic uncertainties. Additionally, the SSH product used in this study has already been denoised using a neural network approach [66]. However, the potential impacts of the denoising procedure on the derived fields warrant further investigation.

SWOT measurement errors may potentially affect our statistical results, but recent validations indicate high precision of SWOT observations. Validation by Wang et al. [67] reported a mean absolute difference of 0.44 ± 0.34 cm for spatial scales below 100 km, with over 70% of the differences falling below 0.6 cm. Another validation using a purely geometric approach [68] found SWOT measurement errors ranging from 0.18 to 0.7 cm at the 10 km spatial scale, comparable to the typical size of mesoscale eddies along the Antarctic coast. Based on these validations, we adopted a threshold excluding eddies with absolute amplitudes less than 0.6 cm to ensure robust results (Fig. 2, Fig. S9 and Fig. S10). As a result, the detected eddies exhibit clear structures (Fig. 1), supporting the reliability of the eddy statistics presented in this study. Moreover, analysis of vortex Rossby numbers across different cycles consistently distinguishes eddy hotspots from regions of low eddy activity (Fig. S12), providing further evidence that SWOT precision is adequate for reliable eddy detection, as otherwise spurious eddy variability would be expected across all regions.

In the SWOT V3.0 data product, SSH is provided in regions where the sea ice concentration is below approximately 70%. This threshold may introduce uncertainties in areas with relatively high ice coverage. To reduce the influence of sea ice, we apply an additional mask based on the observed sea ice concentration from the SWOT Level-2 product. Specifically, SSH fields are retained only where the ice concentration is below 40%. This threshold represents a compromise between maximizing SSH coverage along the Antarctic coast and minimizing potential errors caused by sea ice contamination. We further note that the spatial distribution of detected eddies remains robust across a range of ice concentration thresholds, indicating that our main findings are not sensitive to the specific choice of this criterion.

Due to the larger Rossby deformation radius in the open Southern Ocean, where eddy scales are accordingly larger, eddies cannot be effectively detected using a single 50 km-wide swath. Therefore, we mask the regions with ocean bathymetry deeper than 3500 m to exclude most of the open Southern Ocean. In addition, we also mask some open ocean areas where the bathymetry is shallower than 3500 m, including northeast of the Antarctic peninsula (50°W to 0°, 65°S to 60°S), north of the Lazarev Sea (0° to 10°E, 68°S to 60°S), northeast of the Prydz Bay (70°E to 90°E, 64°S to 60°S), north of the Ross Sea (150°E to 120°W, 67°S to 60°S) and (179°E to 150°W, 70°S to 60°S), and northwest of the Antarctic Peninsula (80°W to 63°W, 62°S to 60°S). Additionally, open ocean regions in the western Ross Sea (178°W to 150°W) with bathymetry deeper than 2000 m are excluded. These masks allow us to focus our study on the Antarctic marginal seas.

**Kinetic energy and vorticity calculation:**

Geostrophic velocity and vorticity are computed from SSH using a second-order central difference method, without additional filtering. Because the SWOT grid spacing is comparable to the local Rossby deformation radius in Antarctic coastal regions, dynamics at these scales are expected to be more strongly geostrophic than in the open ocean [69]. However, small scale SSH errors may propagate into the derived velocity and vorticity fields, introducing uncertainties and underscoring the need for further methodological improvements [70]. In our analysis, kinetic energy (KE) and vorticity are derived from absolute dynamic topography (ADT), obtained by combining the mean dynamic topography (MDT) [71] with SSH anomalies. Absolute KE serves as an indicator of regions where the Antarctic Slope Current is strong and may therefore contribute significantly to eddy generation. In addition, the MDT is spatially smooth, and its contribution to KE and vorticity is negligible.

The kinetic energy variations associated with velocity differences between successive SWOT cycles (hereafter referred to as the velocity-difference kinetic energy, VDKE) are defined as

$$VDKE=\frac{1}{2}\left( {\Delta u}^{2}+{\Delta v}^{2} \right), (3)$$

where $\Delta u$ and $\Delta v$ denote the differences in the velocity components. To quantitatively compare VDKE with KE across successive SWOT cycles, we compute their spatial integrals. The results indicate that the integrated VDKE is slightly larger than the KE in adjacent cycles (Fig. S15). Given that absolute KE includes mean components, such as slowly evolving background flows and quasi-stationary small gyres along the Antarctic coast [22,72], the magnitude of VDKE over successive 21-day SWOT cycles indicates substantial temporal variability in oceanic features.

**Mesoscale eddy identification:**

Mesoscale eddies are detected from the SWOT observations using a commonly used eddy identification method based on the SSH anomaly contours [32,42,73]. Contours are extracted from the SWOT swaths at an interval of 0.1 cm. The average of the innermost closed contour is regarded as the eddy center, and the outermost closed contour that contains no more than one eddy center is regarded as the eddy edge. The eddy radius is defined as the radius of a circle that has the same area as the eddy, and the eddy amplitude is defined as the SSH difference between the eddy center and its edge (eddy center minus eddy edge). Since the SWOT swath is only 50 km wide, it may not capture the full eddy structure, and some of the detected eddies may correspond only to the inner cores of the actual eddies (Fig. S1). As a result, the detected eddy radius may be smaller than the true eddy size. In contrast, we excluded eddies with amplitudes smaller than 0.6 cm to reduce potential observational errors, which substantially reduces the number of smaller scale eddies in the analysis.

**Numerical model setup:**

Currently, there are no realistic circum-Antarctic models that have sufficient resolutions to resolve mesoscale eddies (~ 1 km), except the LLC4320 [12,74]. However, the LLC4320 does not have ice shelf cavities, limiting its application for the present study. To circumvent this limitation of comprehensive models, we set up an idealized high-resolution model to test whether a single driver, such as ice shelf meltwater plumes or DSW export, is able to produce eddies comparable to those observed by SWOT. We use the Massachusetts Institute of Technology General Circulation Model (MITgcm) [75,76] because of its fidelity in simulating meltwater plumes near steep ice shelf fronts [77]. Two model setups are employed, both with a horizontal resolution of 1 km and vertical resolutions of 10 m in the upper 300 m, 15 m between 300 and 900 m depths, and gradually increasing layer thickness to 90 m at 2500 m depth. The lateral boundary conditions are Orlanski radiation conditions, with a relaxation layer of 30 grid cells, and the timescales for inner and outer relaxation are 10 and 0.5 days, respectively. We use a 7th-order non-linear tracer advection scheme, and control grid-scale energy and enstrophy via a biharmonic Leith viscosity with dimensionless vortical and solenoidal viscosities both set to 2.0. The hydrostatic approximation is used throughout these experiments. The initial conditions (Fig. S6) are drawn from in situ observations [19].

For the ice shelf melting experiments, the model domain is 800 x 400 km, with a 300 x 100 km ice shelf embedded in the embayment [78] (Fig. 3B). The ice shelf front is 200 m thick, increasing linearly to 500 m at the south boundary (Fig. 3E). The continental shelf is 500 m deep, sloping linearly to 700 m at the southern boundary under the ice shelf (Fig. 3E). The open ocean has a maximum depth of 2500 m, connected to the continental shelf by a 50 km-wide continental slope prescribed by a hyperbolic tangent function. To simplify the dynamics, the bathymetry is uniform in the zonal direction, and the stratification has no horizontal variations. There is no atmospheric forcing nor background flow, so the buoyancy change due to meltwater from the ice shelf cavity is the sole forcing mechanism. As the observational period coincides approximately with the annual sea ice minimum, we do not include sea ice in this model. We conducted a set of sensitivity experiments to examine the effects of different ice shelf melt rates. This was achieved by adjusting the basal drag coefficient of the ice shelf. The corresponding drag coeffeicents are 0.00001, 0.000025, 0.00005, 0.0001, 0.00025, 0.0005, 0.001, and 0.004 for the experiments with increasing melt rates shown in Fig. 3C. All the experiments reached an approximate equilibrium within half a year (Fig. S7). Following this, an additional 180 days were simulated to calculate EKE and thus quantify the eddy intensity.

For the DSW export experiments, the model domain is 800 x 600 km, consisting of a 300 x 300 km embayment (Fig. 4B) and a flat abyssal ocean with a depth of 2500 m. The continental slope is 50 km wide, prescribed via a hyperbolic tangent function. A trough is located on the western portion of the embayment to steer the DSW export. Its depth increases from 600 m at the shelf break to 900 m at the south boundary. Except for the trough, the bathymetry is uniform in the zonal direction, and the initial stratification has no variations in the horizontal direction. There is no atmospheric forcing, background flow and sea ice component. The DSW is restored to a constant temperature of -1.8 ℃, with salinity varying from 34.65 to 35 psu in different simulations to investigate the influence of different DSW densities. The horizontal area of restoring is a semi-circle with a radius of 50 km (Fig. 4B). To ensure that surface eddy signals originate from the DSW export in the ocean interior, we only restore the water column below 300 m depth (Fig. 4E), so the meridional DSW export in the model represents a relatively weak case due to the relatively small meridional pressure gradient [79]. All the experiments reached approximate equilibrium within 300 days (Fig. S8), after which additional 200 days were simulated to calculate EKE as a measure of eddy intensity.

**Eddy statistics and vorticity probability density distributions in the model:**

For the eddy statistics derived from the model, we select the same regions as those shown in Figs. 3B and 4B and use the same 21-day time window to match the SWOT observations, with the central date corresponding to those in Figs. 3B and 4B. The same data selection is also used to compute the vorticity probability density distributions (Fig. S3B). To enable a direct comparison with the 50 km SWOT swath observations, the model SSH fields are further subsampled into 50 km wide swaths, and the same eddy detection and statistical methods are applied. As shown in Fig. S11, the proportion of small-amplitude eddies (0.6–1 cm) is lower, the eddy radii are larger, and the overall eddy strength is weaker compared to those observed by SWOT (Fig. 2). These discrepancies probably arise from several factors. First, the model output represents daily mean SSH, whereas SWOT provides instantaneous snapshots. Temporal averaging may suppress extreme SSH signals, producing smoother eddy field and larger inferred eddy size. Second, limitations associated with model resolution, domain, bathymetry and simplified forcing may further constrain the model’s ability to fully capture realistic ocean dynamics. In particular, DSW is restored only below 300 m depth (Fig. 4E), which limits the available potential energy and, consequently, eddy generation. Further work is needed to assess the influence of these factors on the eddy representation in model simulations.

**Calculating EKE for the model results:**

We calculate the EKE after the model reaches quasi-equilibrium. The EKE is defined as:

$$\mathrm{EKE}=\frac{1}{2}{|\boldsymbol{u}-\bar{\boldsymbol{u}}|}^{2}, (4)$$

where $\boldsymbol{u}=(u,v)$, and the overbar denotes an average over the last 180 days and 200 days for the ice shelf melting (Fig. S7) and DSW export (Fig. S8) experiments, respectively.

**Accuracy of vortex Rossby number derived from the geostrophic balance relation:**

To assess the reliability of vortex Rossby numbers derived from sea surface height via the geostrophic balance, we compare them with values calculated from surface velocity. This evaluation is based on our numerical model. To ensure consistency with the SWOT satellite resolution, we first average our 1 km model output to a 2 km grid. We then compute the root mean square deviation (RMSD) of the vortex Rossby number over the model domain shown in Fig. S5, excluding regions beneath ice shelf cavities:

$$RMSD=\sqrt{\frac{1}{N}\sum_{i=1}^{N} {(R_{o\_flow}^{i}-R_{o\_ssh}^{i})}^{2}}=0.02. (5)$$

Here, $R_{o\_flow}$ and $R_{o\_ssh}$ represent the vortex Rossby number calculated from the surface velocity field and from the sea surface height via the geostrophic balance (Equation 1), respectively.

We also compute the spatial standard deviation of $R_{o_{\_flow}}$ as:

$$R_{o\_std}=\sqrt{\frac{1}{N}\sum_{i=1}^{N} {(R_{o\_flow}^{i}-\bar{R_{{o\_}_{flow}}})}^{2}}=0.10. (6)$$

The overbar indicates spatial average. The ratio between $RMSD$ and $R_{o\_std}$ is approximately 0.2, indicating that the discrepancy is small relative to the spatial variability of the field. This supports the validity of estimating the vortex Rossby number from SSH via the geostrophic balance.

**Normalized EKE vertical distribution:**

For the normalized EKE distribution in vertical direction, referred to as ‘EKE proportion’ in Fig. 3D and 4D, we first calculate the EKE in each 10 m-thick layer, and then normalize it by dividing by the total EKE integrated over the entire water column:

$$p_{i}=\frac{∯ \int_{z_{i}-10}^{z_{i}} \frac{1}{2}(u^{'}+v^{'})dzdydx}{∯ \int_{-H}^{0} \frac{1}{2}(u^{'}+v^{'})dzdydx}, (7)$$

where ($u^{'},v^{'}$) represent the deviations from the time-averaged flow. For the ice shelf melting experiment, the horizontal integration area is shown in Fig. 3B, excluding the ice shelf cavity. For the DSW export experiment, the integration is limited to the trough area on the continental shelf, defined as the region with a maximum depth greater than 500 m and $y\leq300 km$ (Fig. 4B).

**References:**

66. Tréboutte A, Carli E, Ballarotta M et al. KaRIn Noise Reduction Using a Convolutional Neural Network for the SWOT Ocean Products. Remote Sensing 2023;15(8):2183.

67. Wang J, Lucas AJ, Stalin S et al. SWOT Mission Validation of Sea Surface Height Measurements at Sub‐100 km Scales. Geophysical Research Letters 2025;52(11):e2025GL114936.

68. Hay A, Watson C, Legresy B et al. In Situ Geometric Validation of SWOT Satellite Observations in Bass Strait, Australia. Earth and Space Science 2025;12(8):e2025EA004326.

69. Archer M, Wang J, Klein P et al. Wide-swath satellite altimetry unveils global submesoscale ocean dynamics. Nature 2025;640(8059):691–6.

70. Tranchant Y ‐T., Legresy B, Foppert A et al. SWOT Reveals Fine‐Scale Balanced Motions Driving Near‐Surface Currents and Dispersion in the Antarctic Circumpolar Current. Earth and Space Science 2025;12(8):e2025EA004248.

71. Jousset S, Mulet S, Greiner E et al. New Global Mean Dynamic Topography CNES-CLS-22 Combining Drifters, Hydrological Profiles and High Frequency Radar Data. Preprint, Preprints, 3 Dec. 2023.

72. Mizobata K, Shimada K, Aoki S et al. The Cyclonic Eddy Train in the Indian Ocean Sector of the Southern Ocean as Revealed by Satellite Radar Altimeters and In Situ Measurements. JGR Oceans 2020;125(6):e2019JC015994.

73. Chaigneau A, Le Texier M, Eldin G et al. Vertical structure of mesoscale eddies in the eastern South Pacific Ocean: A composite analysis from altimetry and Argo profiling floats. J Geophys Res 2011;116(C11):2011JC007134.

74. Forget G, Campin JM, Heimbach P et al. ECCO version 4: an integrated framework for non-linear inverse modeling and global ocean state estimation. Geosci Model Dev 2015;8(10):3071–104.

75. Marshall J, Hill C, Perelman L et al. Hydrostatic, quasi‐hydrostatic, and nonhydrostatic ocean modeling. J Geophys Res 1997;102(C3):5733–52.

76. Marshall J, Adcroft A, Hill C et al. A finite‐volume, incompressible Navier Stokes model for studies of the ocean on parallel computers. J Geophys Res 1997;102(C3):5753–66.

77. Losch M. Modeling ice shelf cavities in a z coordinate ocean general circulation model. J Geophys Res 2008;113(C8):2007JC004368.

78. Si Y, Stewart AL, Silvano A et al. Antarctic Slope Undercurrent and onshore heat transport driven by ice shelf melting. Sci Adv 2024;10(16):eadl0601.

79. Bowen MM, Fernandez D, Gordon AL et al. Tides regulate the flow and density of Antarctic Bottom Water from the western Ross Sea. Sci Rep 2023;13(1):3873.

**
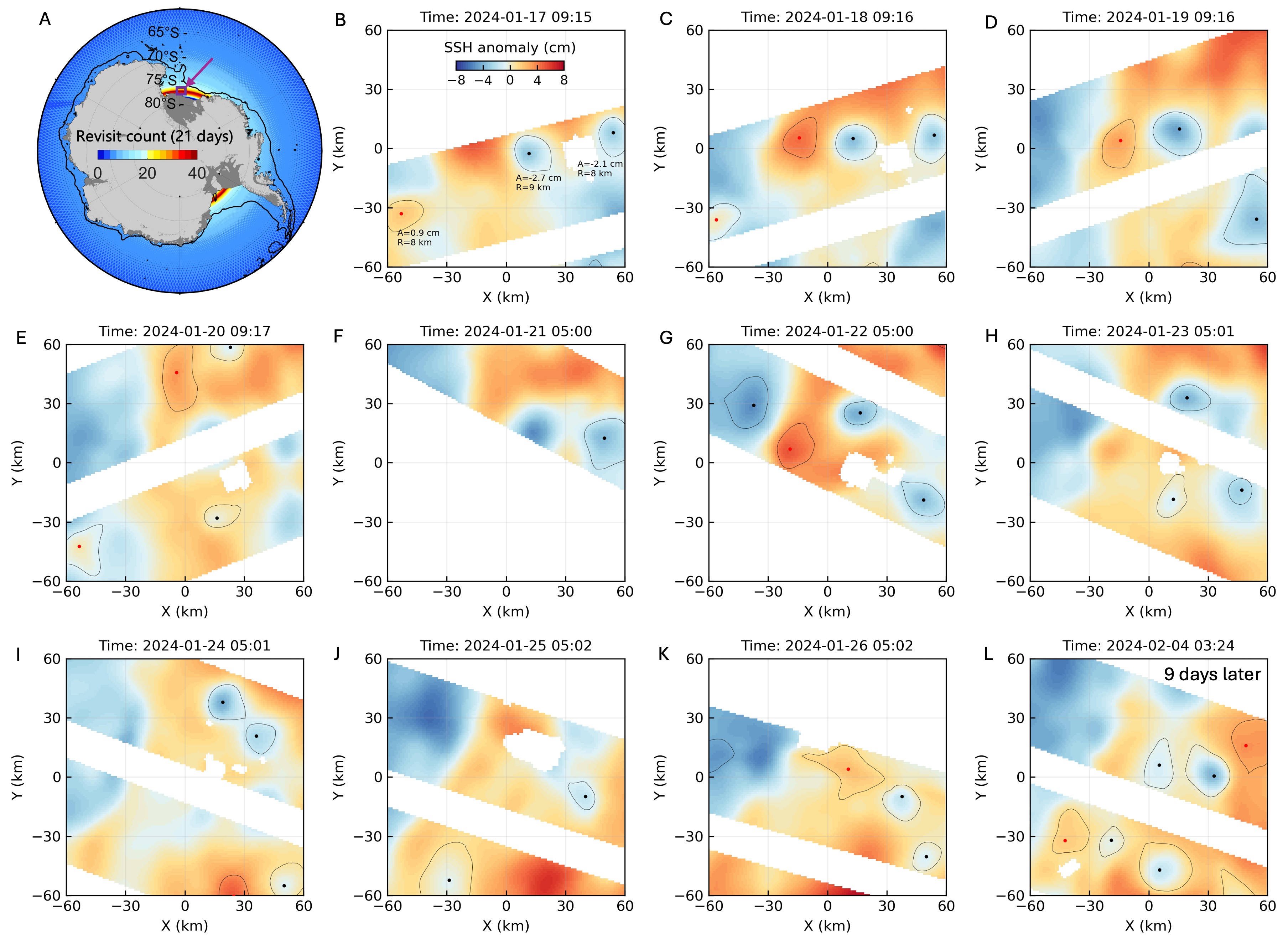
**

**Figure S1. Eddy evolution in the southern Ross Sea.** (A) Number of orbit revisits during a 21-day SWOT cycle, with the rectangle indicating the location of the detection region. (B–L) Near-daily evolution of sea surface height anomaly. Black circles denote eddy boundaries, while black and red dots mark the centers of cyclonic and anticyclonic eddies, respectively. Panel B illustrates examples of eddy amplitude (A) and radius (R). Only eddies with amplitudes exceeding 0.6 cm are shown, consistent with Fig. 2. Note that panel L is 9 days after panel K. All data are projected from geographic coordinates to Cartesian distance coordinates, with the domain centered at 76.8°S, 179.8°W.

**
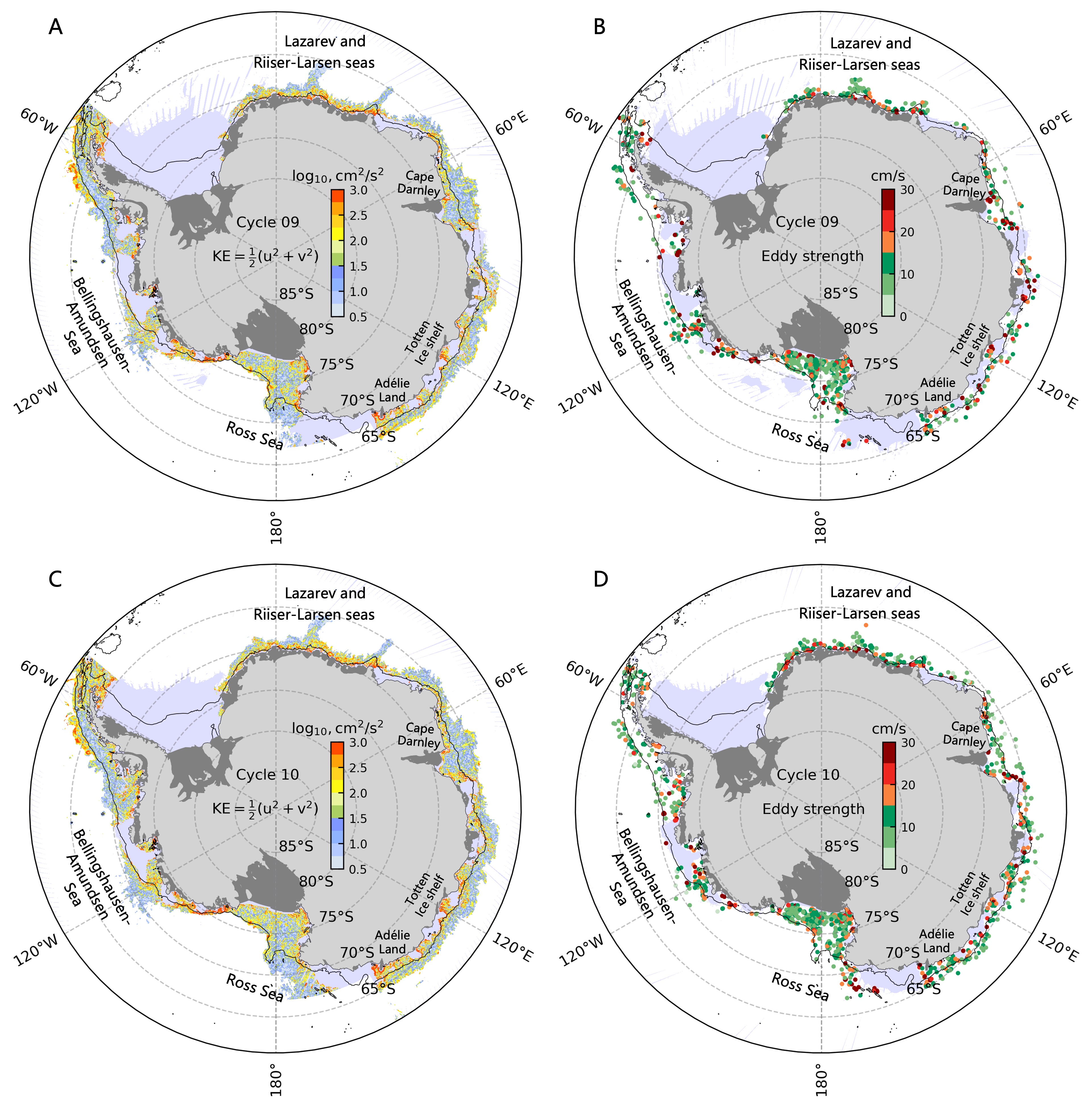
**

**Figure S2. Snapshots of SWOT observations.** Snapshot of kinetic energy (A) and detected eddies (B) for SWOT cycle 09 (January 04 to 25, 2024). (C-D) Similar to panels (A-B), but for cycle 10 (January 25 to February 14, 2024). The black and light purple shading indicate ice shelf and sea ice coverage, respectively. The circum-Antarctic black line indicates the 1000 m isobath.


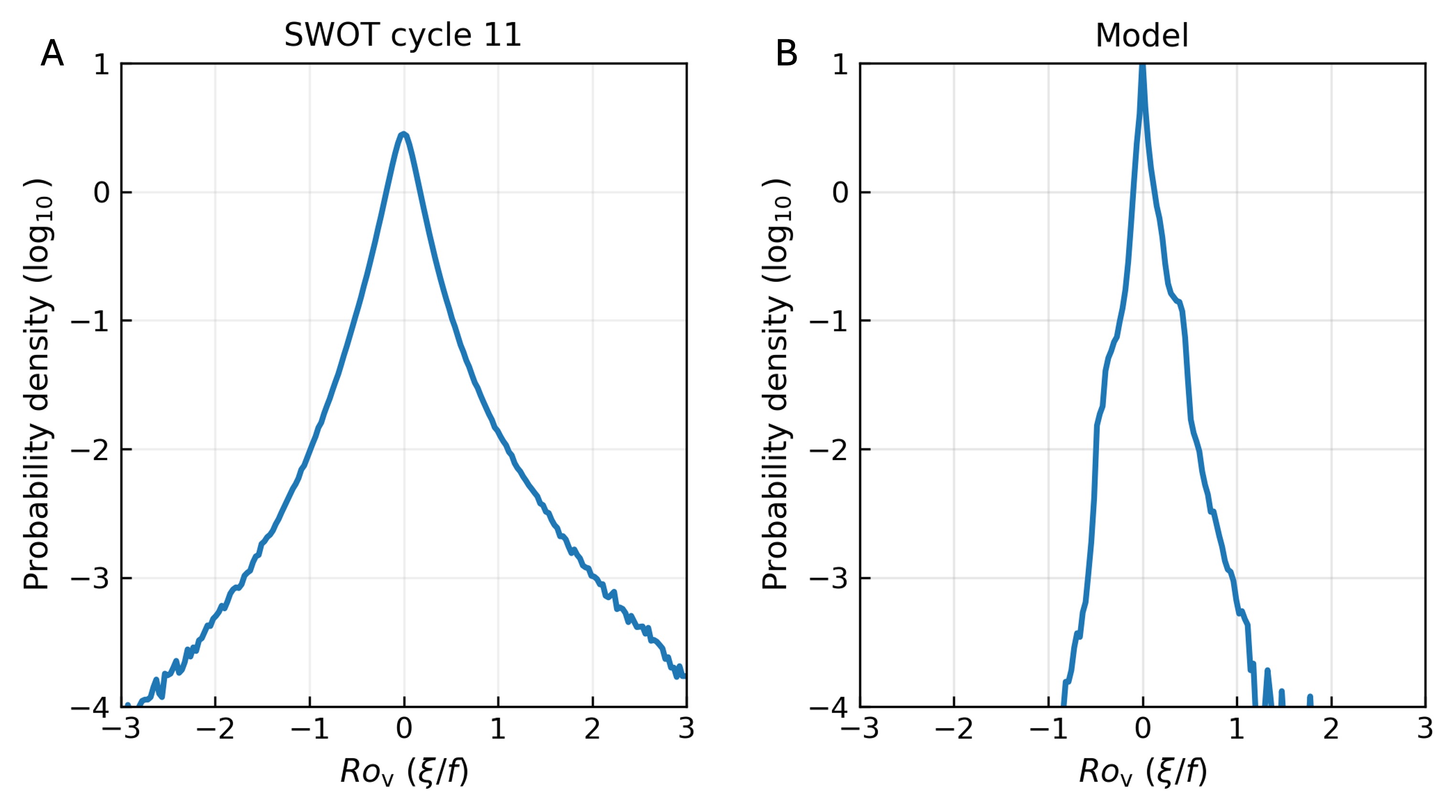


**Figure S3. Probability density functions of the vortex Rossby number derived from SWOT observations (A) and from model simulations (B).** For the observations, we use the SWOT cycle 11 and mask the open ocean regions, consistent with the areas shown in Fig. 1A. For the model results, we select the same regions as in Figs. 3B and 4B and apply a 21-day time window to match the SWOT sampling, with central dates aligned with those figures.


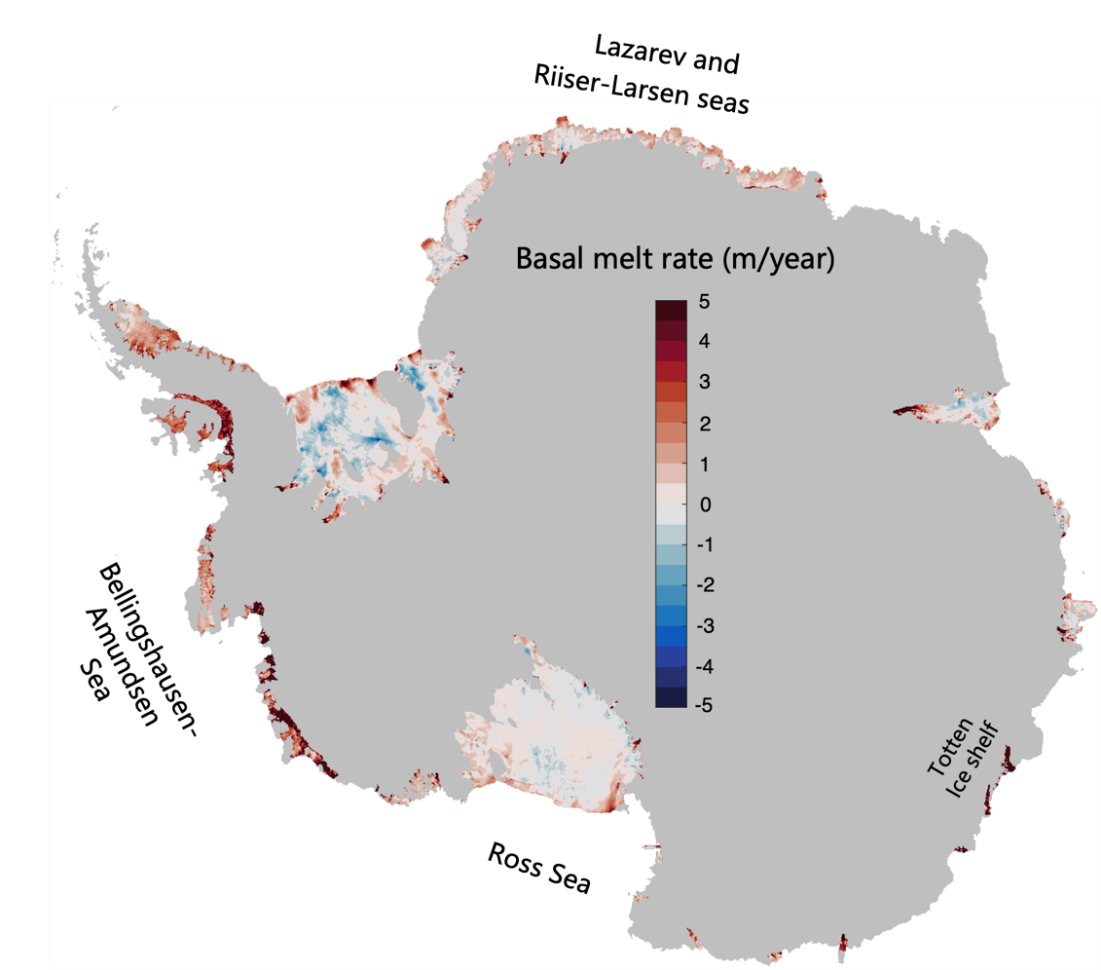


**Figure S4. Ice shelf basal melt rates averaged over 2010-2018 (ref. *3*).** Positive value means melting.


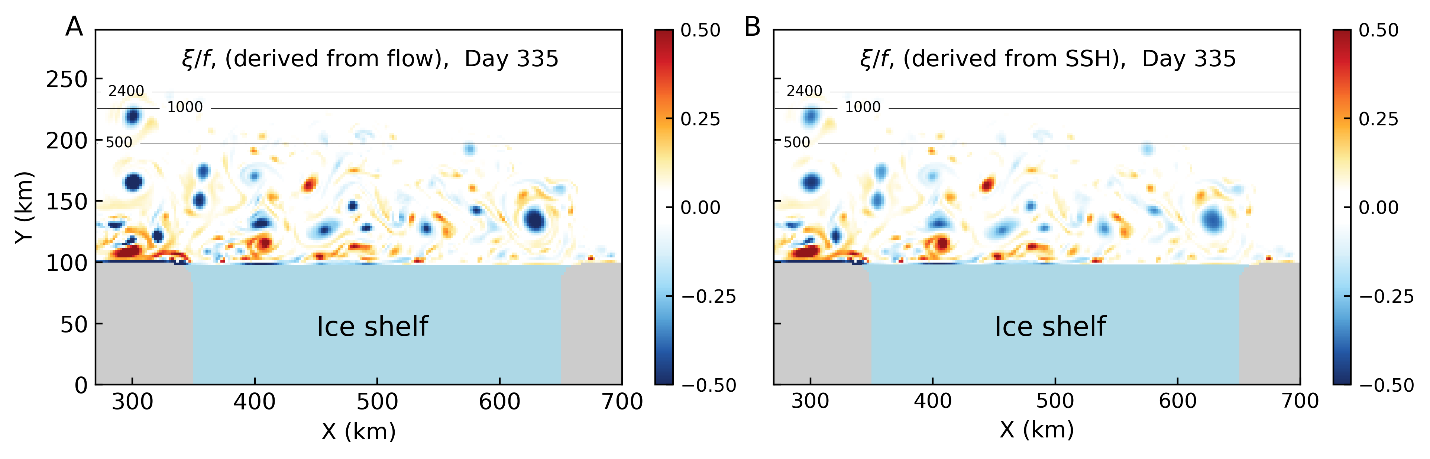


**Figure S5. Comparison of vortex Rossby numbers**. (A) Derived from surface flow, and (B) derived from sea surface height (SSH) via geostrophic balance (Equations 1 and 2). The 1 km model output has been averaged to a 2 km grid to match the resolution of the SWOT product. Contours indicate bathymetric isobaths. The two patterns are highly similar, with a root mean square deviation (RMSD) of 0.02 (see Methods), suggesting that the geostrophic balance method (Equations 1 and 2) is applicable to the Antarctic marginal seas.


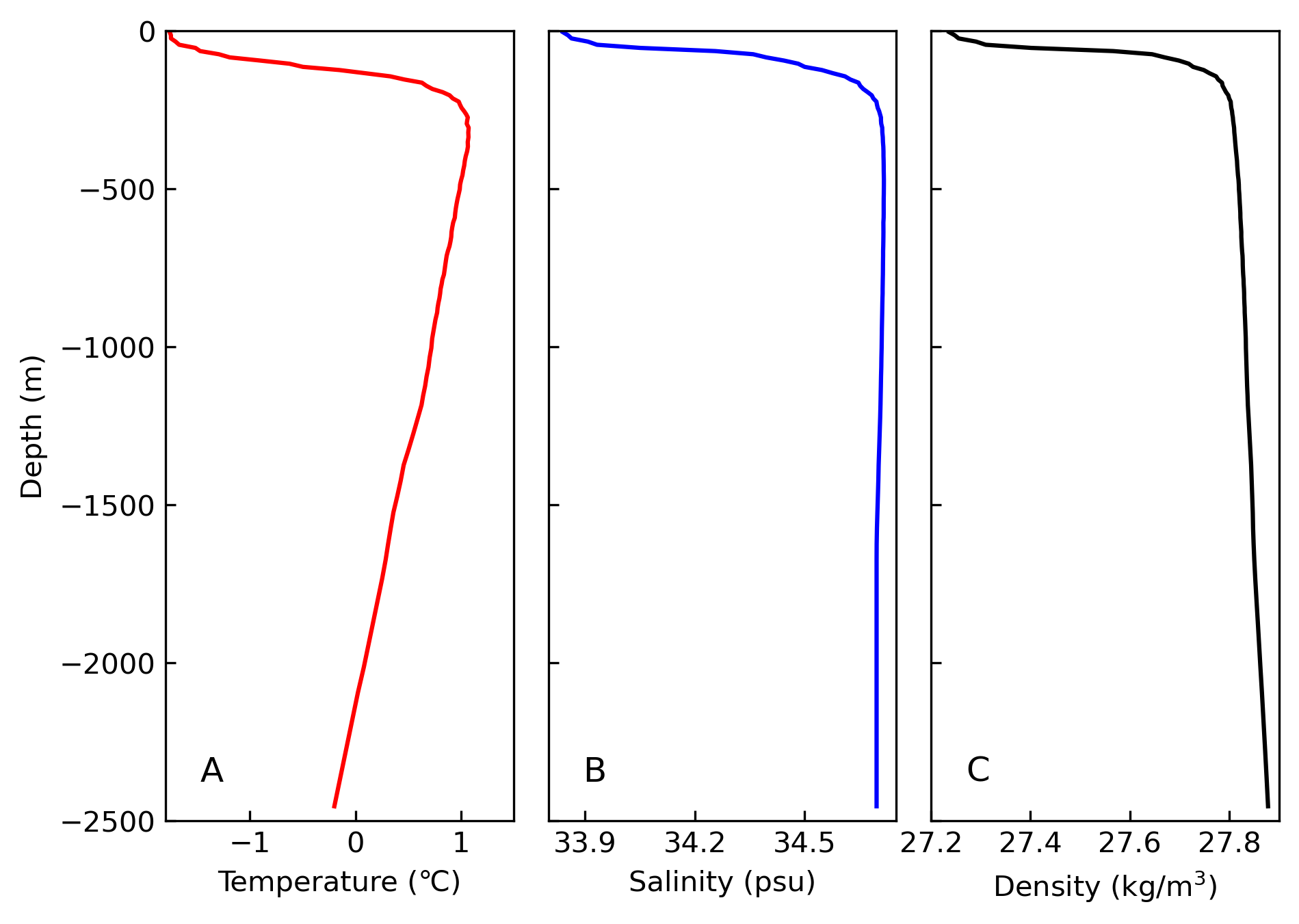


**Figure S6. Model initial conditions.** Profiles of initial temperature (A), salinity (B) and potential density (referenced to 0 dbar) (C). They are horizontally invariant at the beginning of the simulations.


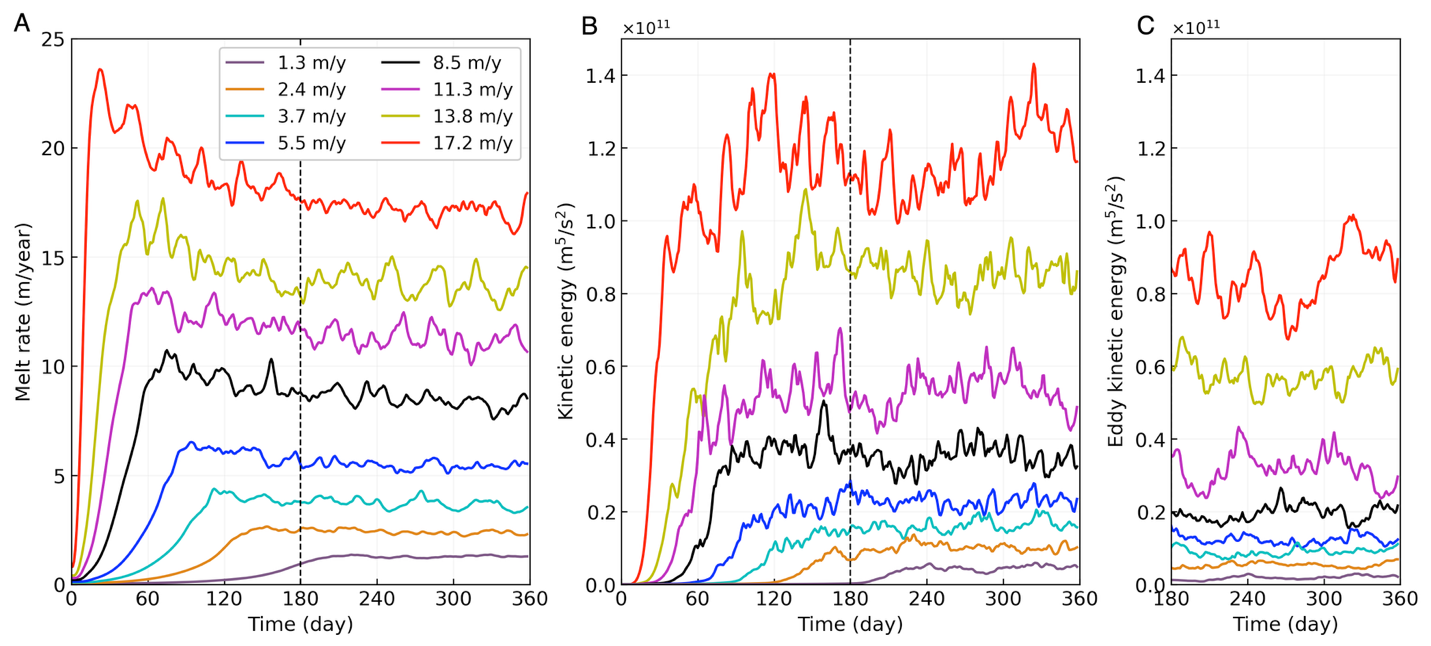


**Figure S7. Time series of sensitivity experiments with different ice shelf melt rates.** (A) Area averaged ice shelf melt rate. The vertical dashed line indicates the moment that we treat as the start of the equilibrium period. (B) Volume-integrated kinetic energy over the regions shown in Fig. 3B, with ice shelf cavity excluded. (C) Volume-integrated eddy kinetic energy in the last 180 days.


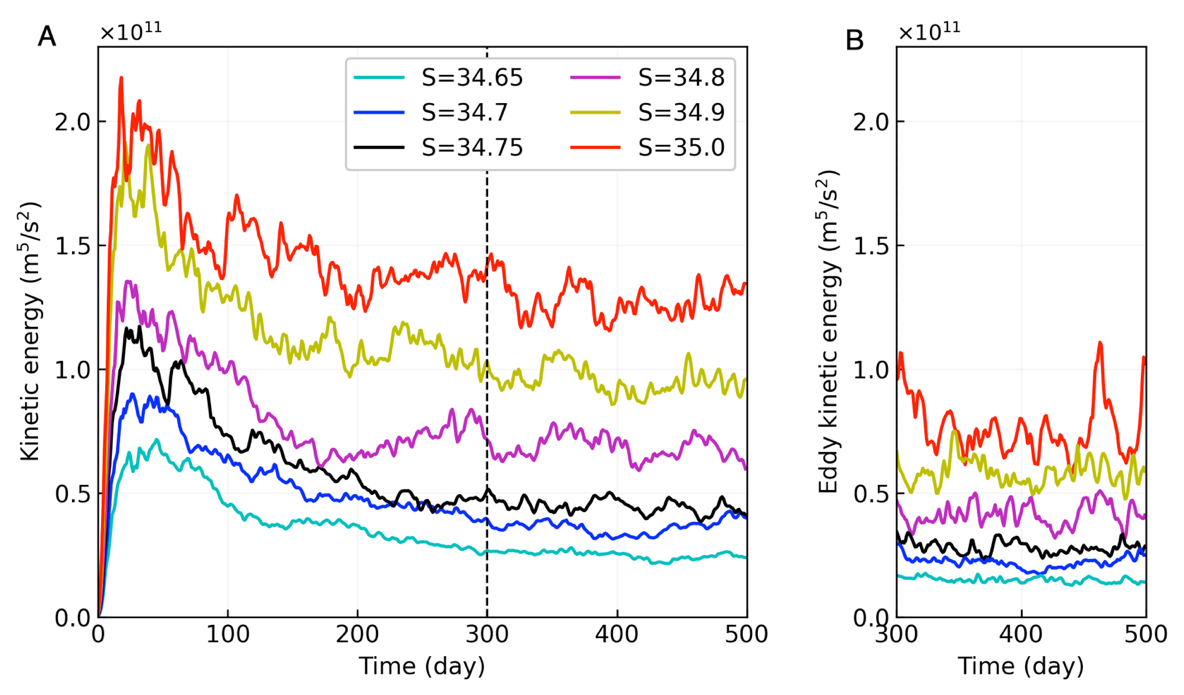


**Figure S8. Time series of sensitivity experiments with different dense shelf water salinities.** (A) Volume-integrated kinetic energy over the trough on the continental shelf as shown in Fig. 4B, with other regions excluded. The vertical dashed line indicates the moment that we treat as the start of the equilibrium period. (B) Volume-integrated eddy kinetic energy over the last 200 days.


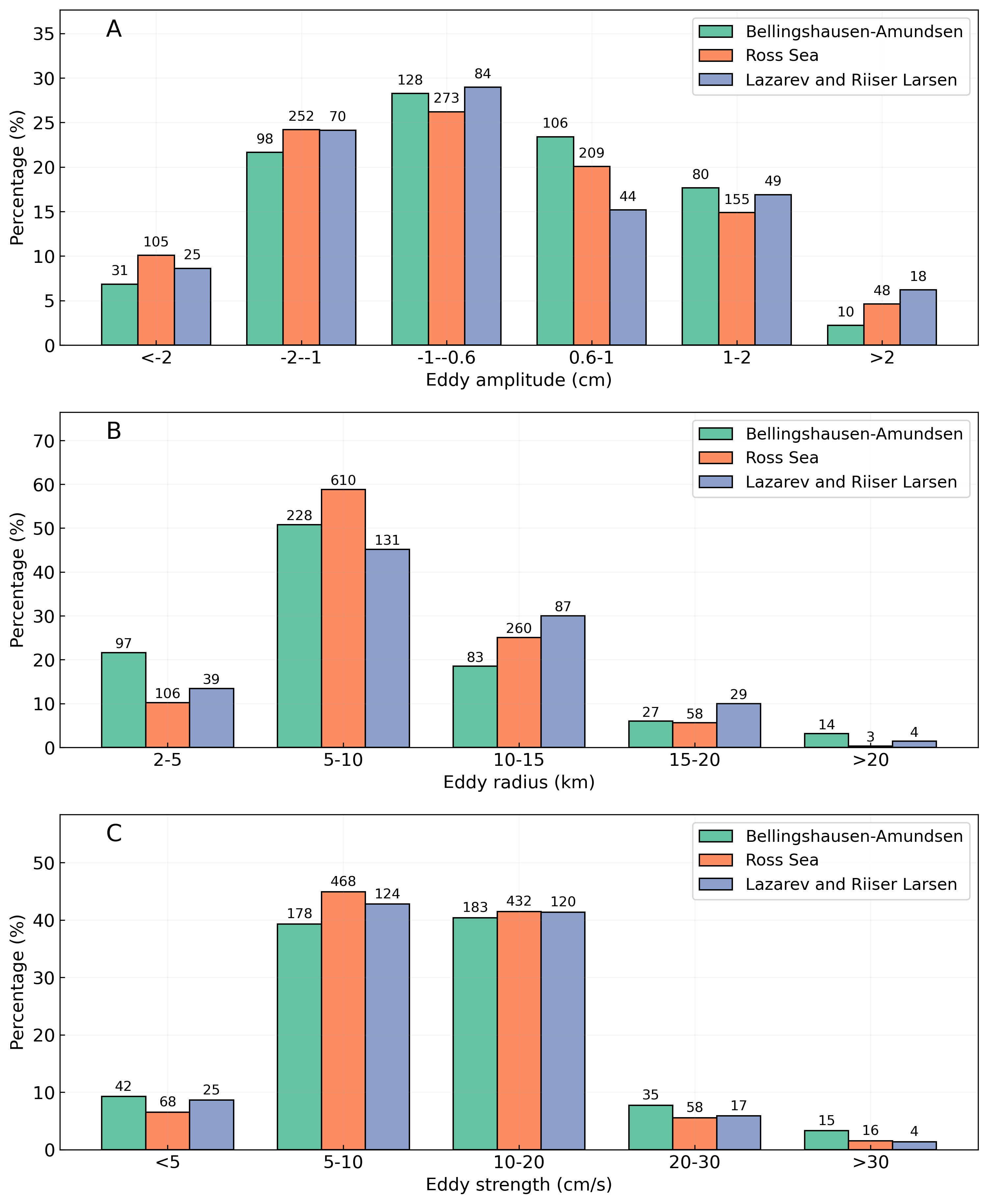


**Figure S9. Eddy statistics in the Bellingshausen-Amundsen Sea sector (120°W to 60°W), the Ross Sea sector (150°E to 150°W), and the Lazarev and Riiser-Larsen Sea sector (10°W to 50°E), respectively.** (A) Eddy amplitude. (B) Eddy radius. (C) Eddy strength. The numbers above each bar represent the counts of detected eddies.


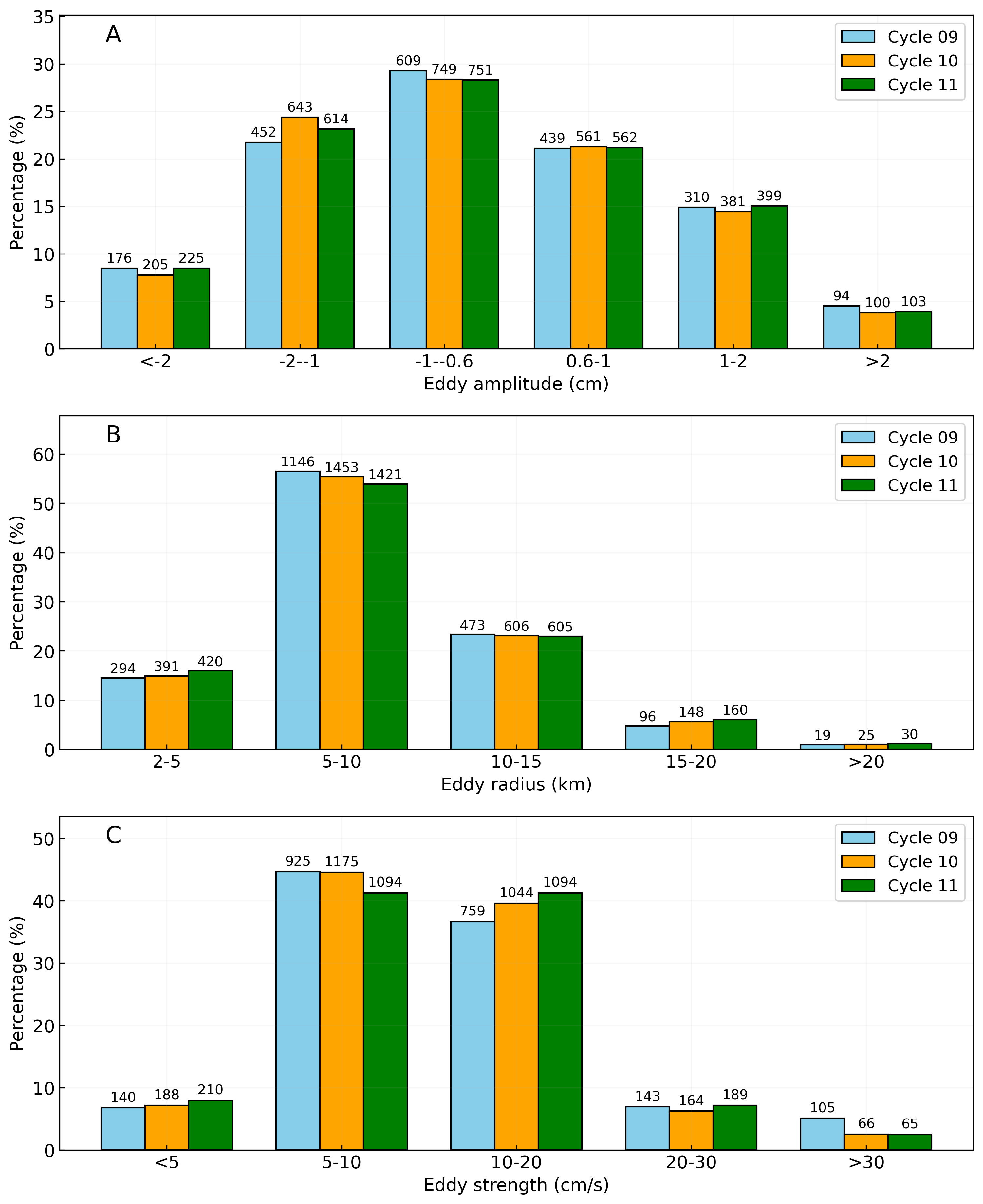


**Figure S10. Pan-Antarctic eddy statistics over different SWOT cycles.** The numbers above each bar represent the counts of detected eddies. Variations in eddy counts between cycles are primarily due to differences in sea ice coverage.


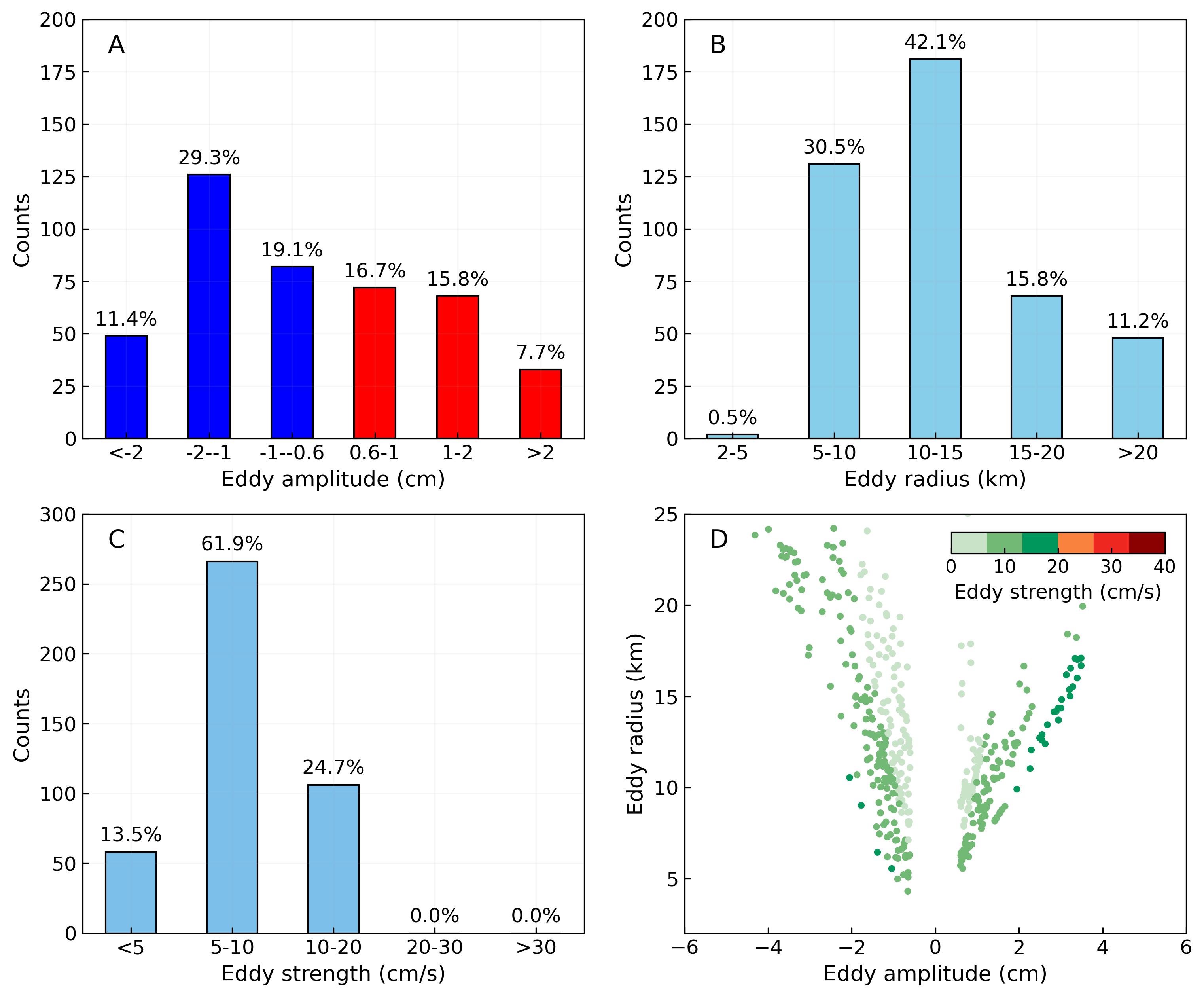


**Figure S11. Similar to Fig. 2, but showing the eddy statistical characteristics derived from the model results (see Methods).** (A) Eddy amplitude. (B) Eddy radius. (C) Eddy strength. (D) Relationship among eddy amplitude, radius, and strength. For the model analysis, the same regions shown in Figs. 3B and 4B are selected, and a 21-day time window is applied to match the SWOT sampling, with central dates corresponding to those in Figs. 3B and 4B, respectively. The model SSH fields are further subsampled into 50 km wide swaths, and eddies with absolute amplitudes smaller than 0.6 cm are excluded, consistent with the SWOT-based analysis.


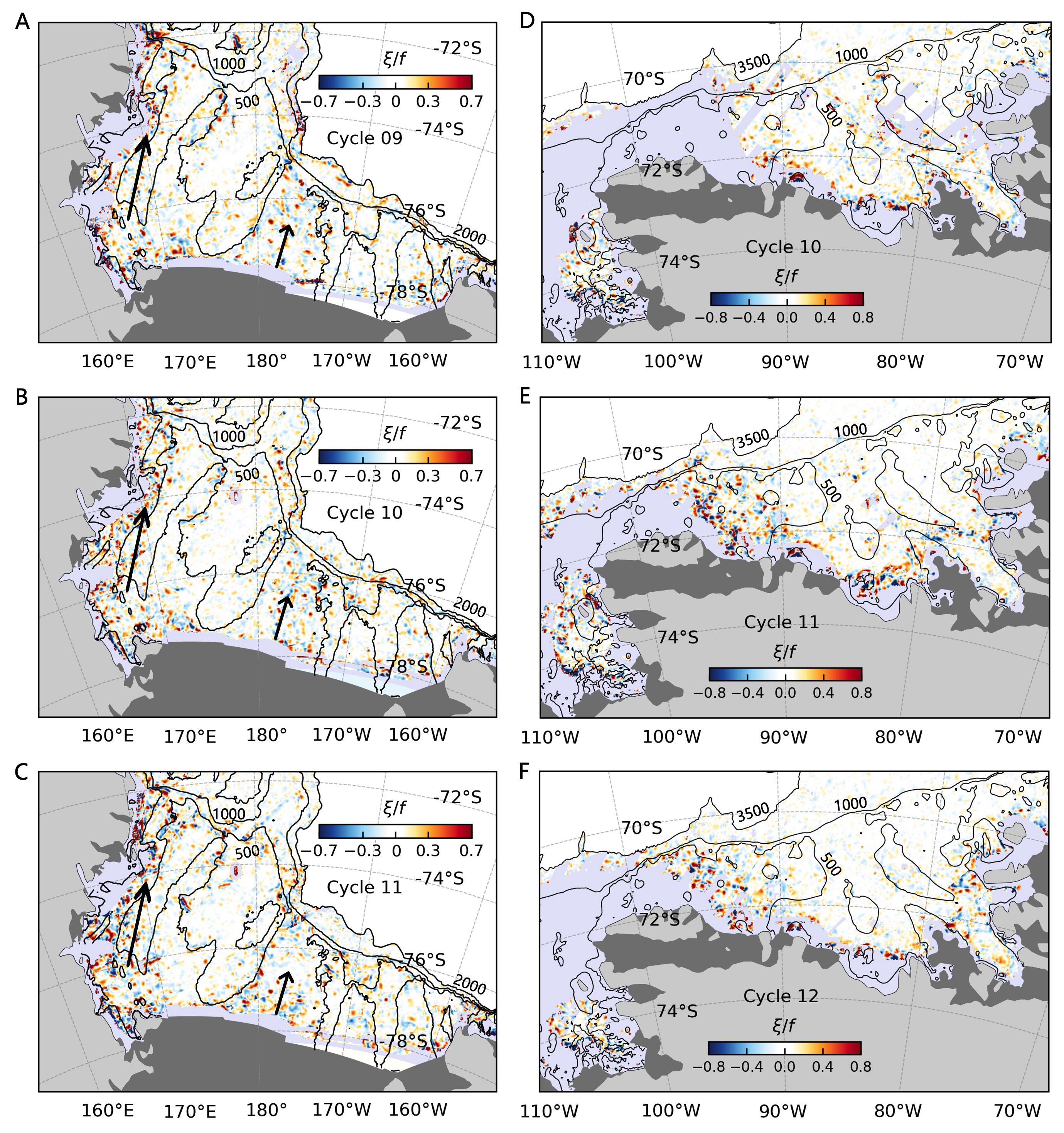


**Figure S12. Eddy variations over three consecutive SWOT cycles.** (A-C) Snapshot of the vortex Rossby number in the Ross Sea from SWOT observations during cycles 09 to 11. Black and light purple shading indicate ice shelf and sea ice coverage, respectively. Black arrows indicate DSW export across the Drygalski Trough (left) and the Glomar Challenger Trough (right), respectively. (D-F) Same as panels A-C, but for the Bellingshausen–Amundsen Sea from SWOT observations during cycles 10-12.


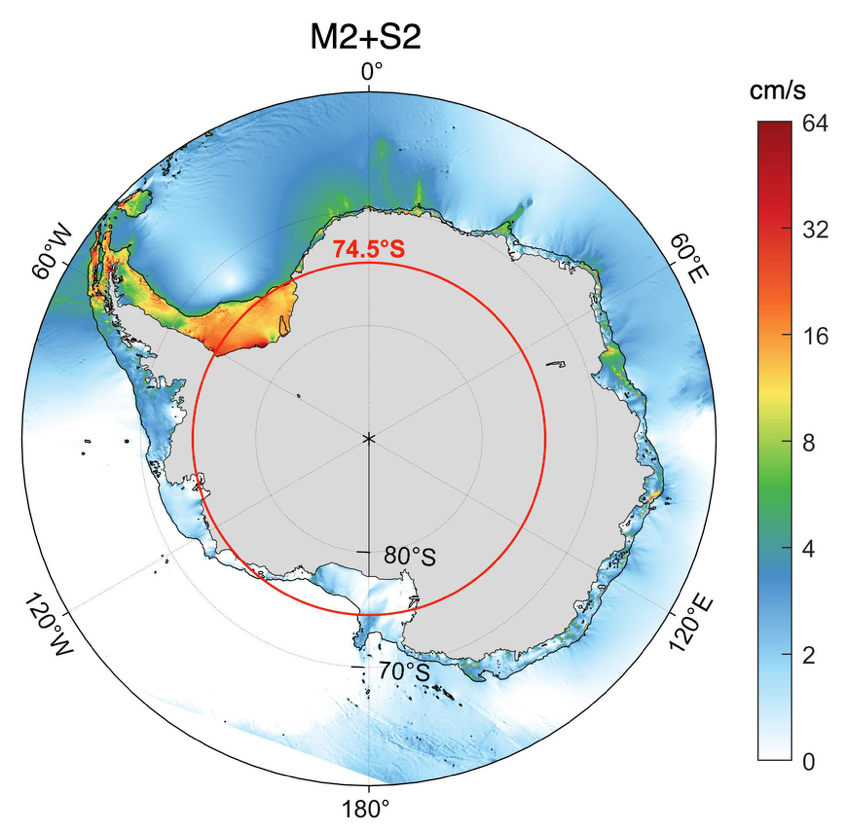


**Figure S13.** **Summation of semidiurnal major axis of the M2 and S2 tidal constituents.** The red solid curve indicates the critical latitude of semidiurnal (M2) tides. Data are derived from the CATS2008 tidal model. The summed major axes can represent the maximum tidal flow speed. As shown, semidiurnal tidal amplitudes are very weak, except in the Weddell Sea, which is covered by sea ice in summer.


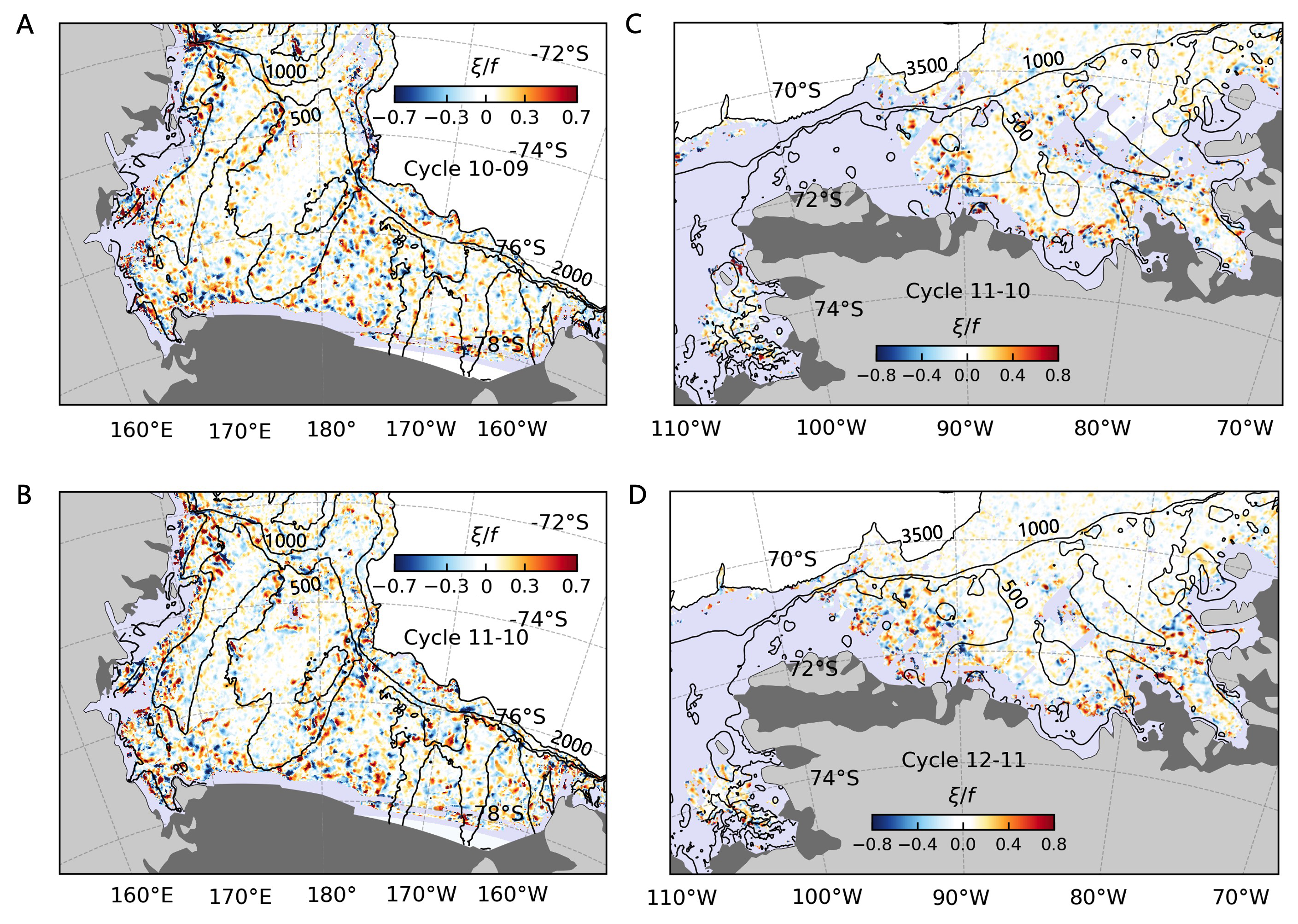


**Figure S14.** **Differences in the vortex Rossby number over successive SWOT cycles.** (A–B) Differences in the vortex Rossby number in the Ross Sea between SWOT cycles 10 and 9, and between cycles 11 and 10, respectively. (C–D) Differences in the vortex Rossby number in the Bellingshausen-Amundsen Sea between cycles 11 and 10, and between cycles 12 and 11, respectively.


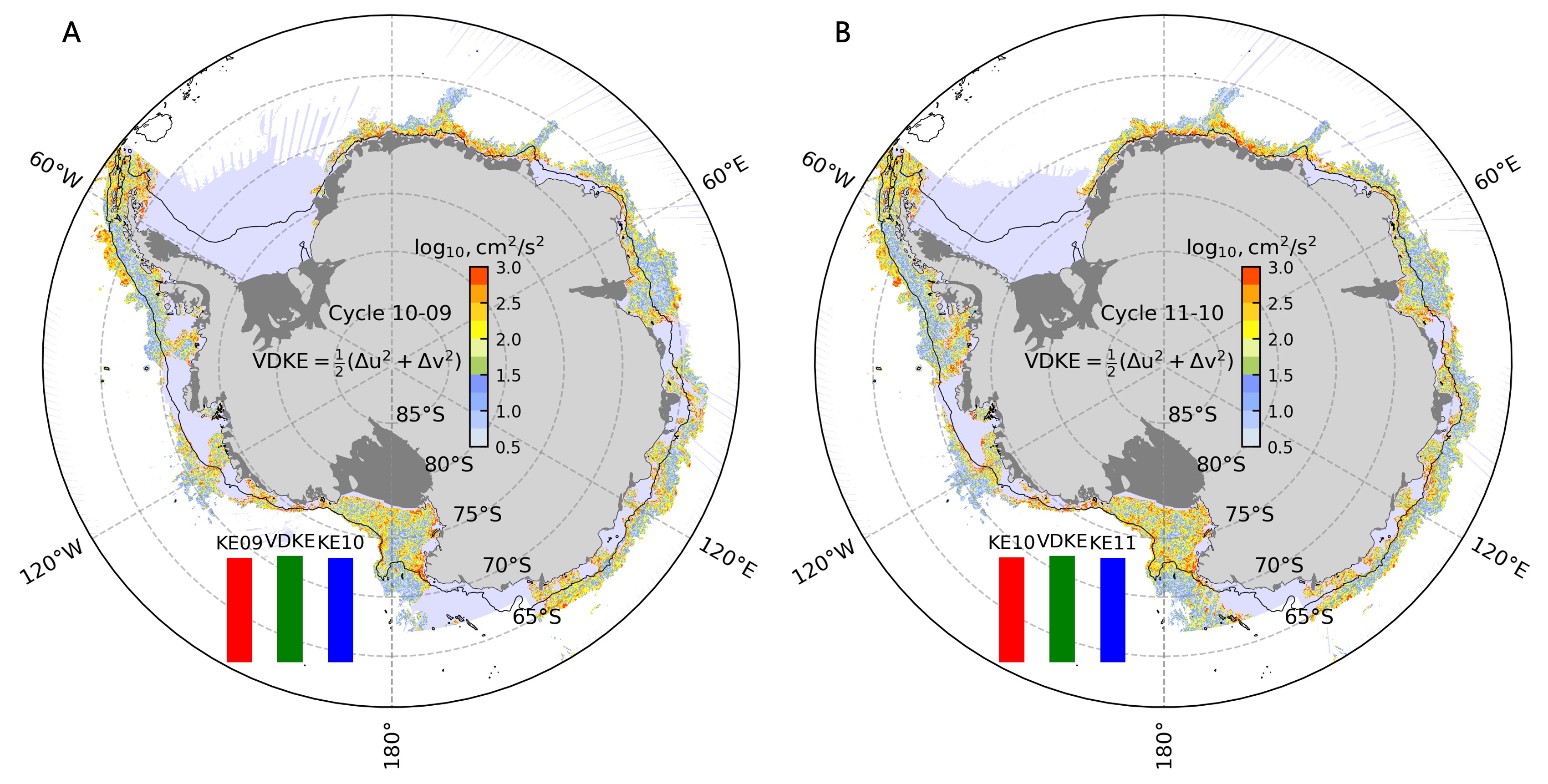


**Figure S15. Kinetic energy variations associated with velocity differences (VDKE) between successive SWOT cycles (see Methods).** (A) VDKE between cycles 10 and 9. (B) VDKE between cycles 11 and 10. The red and blue bars represent the spatially integrated KE for adjacent cycles along the Antarctic margins, while the green bars indicate the corresponding VDKE.


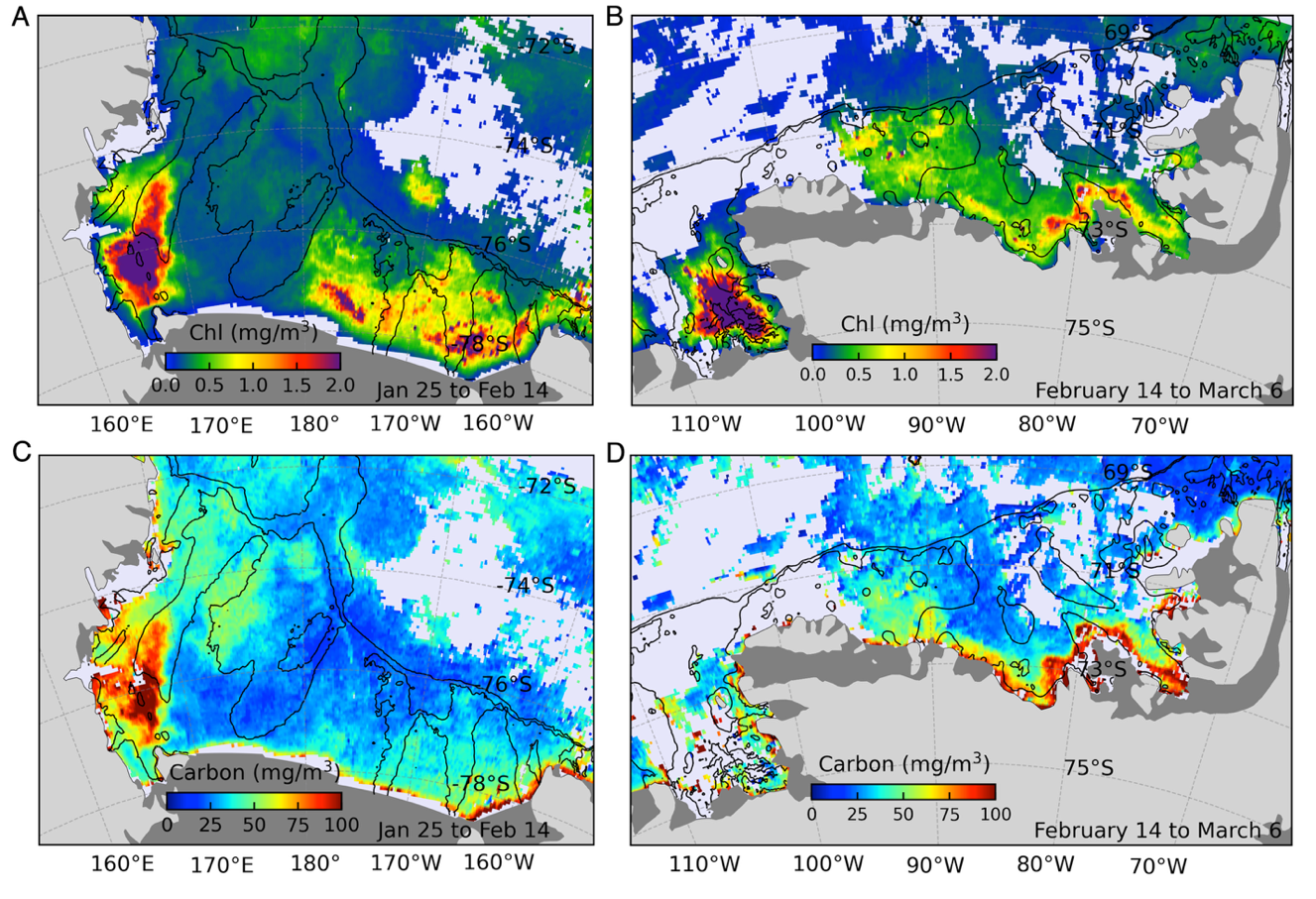


**Figure S16. Chlorophyll-a and phytoplankton carbon concentrations.** (A) Chlorophyll-a concentration averaged over January 25–February 14, 2024, corresponding to SWOT cycle 10 shown in Fig. 4A. Black and light purple shading indicate ice shelf and sea ice coverage, respectively. (B) Chlorophyll-a concentration averaged over February 14–March 6, 2024, corresponding to SWOT cycle 11 shown in Fig. 3A. (C–D) Same as (A) and (B), but for phytoplankton carbon concentration. Chlorophyll-a and phytoplankton carbon data are obtained from the SNPP_VIIRS L3 and L4 daily 4 km products, respectively.
